# Sensitive Detection of Asymptomatic and Symptomatic Malaria with Seven Novel Parasite-Specific LAMP Assays and Translation for Use at Point-of-Care

**DOI:** 10.1128/spectrum.05222-22

**Published:** 2023-05-09

**Authors:** Kenny Malpartida-Cardenas, Nicolas Moser, Felix Ansah, Ivana Pennisi, Diana Ahu Prah, Linda Eva Amoah, Gordon Awandare, Julius Clemence R. Hafalla, Aubrey Cunnington, Jake Baum, Jesus Rodriguez-Manzano, Pantelis Georgiou

**Affiliations:** a Department of Infectious Disease, Faculty of Medicine, Imperial College London, London, United Kingdom; b Department of Electrical and Electronic Engineering, Faculty of Engineering, Imperial College London, London, United Kingdom; c West African Centre for Cell Biology of Infectious Pathogens, Department of Biochemistry, Cell and Molecular Biology, University of Ghana, Legon, Ghana; d Department of Infection Biology, Faculty of Infectious and Tropical Diseases, London School of Hygiene and Tropical Medicine, London, United Kingdom; e Immunology Department, Noguchi Memorial Institute for Medical Research, University of Ghana, Legon, Ghana; f Department of Life Sciences, Faculty of Natural Sciences, Imperial College London, London, United Kingdom; g School of Biomedical Sciences, University of New South Wales Sydney, Sydney, Australia; Hubei University of Medicine

**Keywords:** nucleic acid amplification, malaria, diagnostics, point-of-care

## Abstract

Human malaria is a life-threatening parasitic disease with high impact in the sub-Saharan Africa region, where 95% of global cases occurred in 2021. While most malaria diagnostic tools are focused on Plasmodium falciparum, there is a current lack of testing non-P. falciparum cases, which may be underreported and, if undiagnosed or untreated, may lead to severe consequences. In this work, seven species-specific loop-mediated isothermal amplification (LAMP) assays were designed and evaluated against TaqMan quantitative PCR (qPCR), microscopy, and enzyme-linked immunosorbent assays (ELISAs). Their clinical performance was assessed with a cohort of 164 samples of symptomatic and asymptomatic patients from Ghana. All asymptomatic samples with a parasite load above 80 genomic DNA (gDNA) copies per μL of extracted sample were detected with the Plasmodium falciparum LAMP assay, reporting 95.6% (95% confidence interval [95% CI] of 89.9 to 98.5) sensitivity and 100% (95% CI of 87.2 to 100) specificity. This assay showed higher sensitivity than microscopy and ELISA, which were 52.7% (95% CI of 39.7 to 67%) and 67.3% (95% CI of 53.3 to 79.3%), respectively. Nine samples were positive for *P. malariae*, indicating coinfections with P. falciparum, which represented 5.5% of the tested population. No samples were detected as positive for P. vivax, *P. ovale*, P. knowlesi, or P. cynomolgi by any method. Furthermore, translation to the point-of-care was demonstrated with a subcohort of 18 samples tested locally in Ghana using our handheld lab-on-chip platform, Lacewing, showing comparable results to a conventional fluorescence-based instrument. The developed molecular diagnostic test could detect asymptomatic malaria cases, including submicroscopic parasitemia, and it has the potential to be used for point-of-care applications.

**IMPORTANCE** The spread of Plasmodium falciparum parasites with *Pfhrp2*/*3* gene deletions presents a major threat to reliable point-of-care diagnosis with current rapid diagnostic tests (RDTs). Novel molecular diagnostics based on nucleic acid amplification are needed to address this liability. In this work, we overcome this challenge by developing sensitive tools for the detection of Plasmodium falciparum and non-P. falciparum species. Furthermore, we evaluate these tools with a cohort of symptomatic and asymptomatic malaria patients and test a subcohort locally in Ghana. The findings of this work could lead to the implementation of DNA-based diagnostics to fight against the spread of malaria and provide reliable, sensitive, and specific diagnostics at the point of care.

## INTRODUCTION

Human malaria is a life-threatening parasitic disease that affected an estimated 247 million people worldwide in 2021. In 2020, malaria deaths increased by 10% compared to in 2019 and declined slightly to 619,000 deaths in 2021 (1, 2). There is an unmet need to implement point-of-care (POC) digital diagnostics for the detection of Plasmodium falciparum and non-P. falciparum cases to reduce the burden of this disease and report progress toward global technical strategic goals. To date, eight species have been identified that infect humans: (i) Plasmodium falciparum, (ii) Plasmodium vivax, (iii) Plasmodium ovale *wallikeri*, (iv) Plasmodium ovale *curtisi*, (v) Plasmodium malariae, (vi) Plasmodium knowlesi, (vii) Plasmodium cynomolgi, and (viii) *Plasmodium simium*. The first five are specific to humans, with *P. ovale wallikeri* and *P. ovale curtisi* considered distinct sympatric *Plasmodium* species that can only be differentiated at the DNA level (3). The last three species, P. knowlesi, P. cynomolgi, and *P. simium*, have been known to infect primate hosts. However, natural transmission by anophelines from primates to humans has recently been reported, and malaria is considered a zoonotic disease in this instance (4).

Current methods for malaria diagnostics include microscopy, antigen detection, and nucleic acid amplification. However, species-specific detection could be challenging by microscopy due to the similar morphology of the parasites (5, 6), and the lower levels of parasitemia of non-P. falciparum species may limit their detection with antigen-based methods. Nucleic acid amplification tests (NAATs) are the most promising tools for species-specific malaria diagnostics due to the high specificity and sensitivity they offer (7–9). PCR assays for the detection of P. falciparum, P. vivax, *P. malariae*, *P. ovale*, or P. knowlesi have been reported. (10–15). However, they require long turnaround times or the use of expensive primer modifications and fluorophores (16). In addition, these methods require expensive, sophisticated equipment and trained/skilled personnel, which is hampering their translation to the POC. As an alternative to PCR, loop-mediated isothermal amplification (LAMP) is a promising candidate for molecular testing outside the laboratory (17). LAMP species-specific assays have been reported mostly targeting the conserved 18S rRNA gene or mitochondrial genes (18–25). However, targeting conserved genes increases the likelihood of interspecies cross-reactivity.

**FIG 1:**
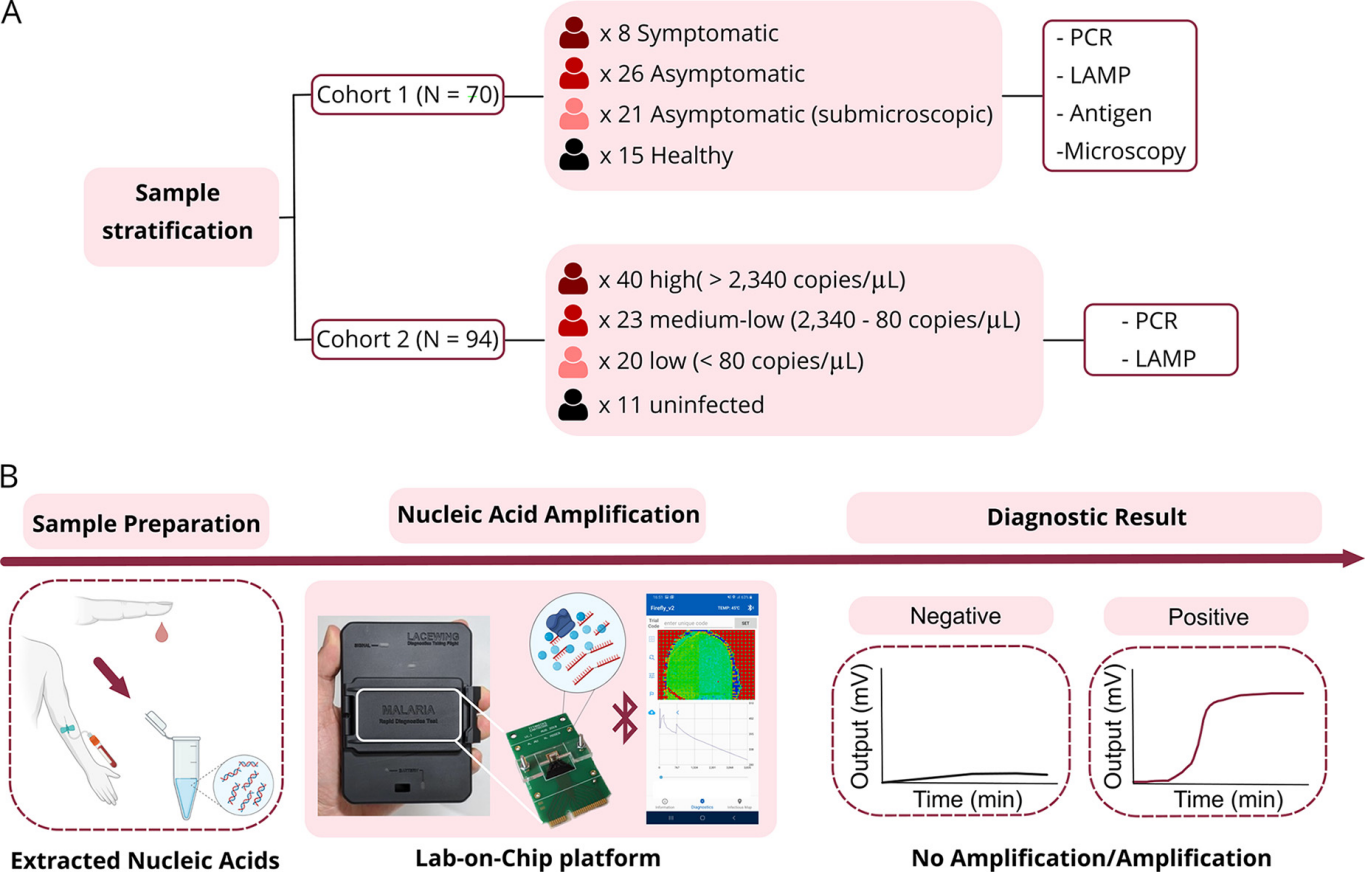
Schematics of the conducted study. (A) Data stratification of the studied cohorts and methods used in this work. (B) Workflow for lab-on-chip diagnostics: nucleic acid extraction from blood, nucleic acid amplification using the lab-on-chip platform, real-time data visualization through a smartphone application, and diagnostic outcome.

In this work, we report seven species-specific LAMP assays for the discrimination of human-infective *Plasmodium* species. Their performance was evaluated with 164 clinical samples from Ghana using TaqMan quantitative PCR (qPCR) as a reference and also performing traditional diagnostic methods, such as microscopy and antigen-based detection. Translation into POC diagnostics was achieved by combining the LAMP assays with our in-house-developed lab-on-chip (LoC) platform called Lacewing. A schematic outline of the study conducted is presented in Fig. 1.

## RESULTS

### LAMP assays for the detection of human-infective *Plasmodium* species.

LAMP assays were designed to discriminate seven human-infective *Plasmodium* species with high sensitivity and specificity: (i) *P. ovale wallikeri* and *curtisi* (duplex, LAMP-PoRBP2), (ii) *P. malariae* (LAMP-PmK13), (iii) P. vivax (LAMP-PvAT2), and (iv) P. cynomolgi (LAMP-PcyAT2). Assays for the specific detection of P. falciparum (26), P. knowlesi (25), and *Plasmodium* pan-genus (23) were included from previous studies. Sequences of selected target genes were collated from PlasmoDB and NCBI (sequences are provided in Table S1, and the accession numbers are in Table S2 in the supplemental material). The LAMP-PoRBP2 assay consisted of a duplex assay targeting a conserved region of the reticulocyte binding protein 2 (RBP2) gene, which is only present in *P. ovale* and P. vivax (27). If the duplex assays are used independently, they can discriminate the two species based on differential time-to-positive (TTP) values (Fig. S1). The LAMP-PmK13 assay was designed based on the *kelch 13* gene, the same gene used for P. falciparum detection (26), leveraging the differences due to their high divergence (27). Lastly, the LAMP-PvAT2 and LAMP-PcyAT2 assays were designed using α-tubulin 2 ortholog genes based on the numerous mismatches found with respect to other *Plasmodium* species (18).

**FIG 2:**
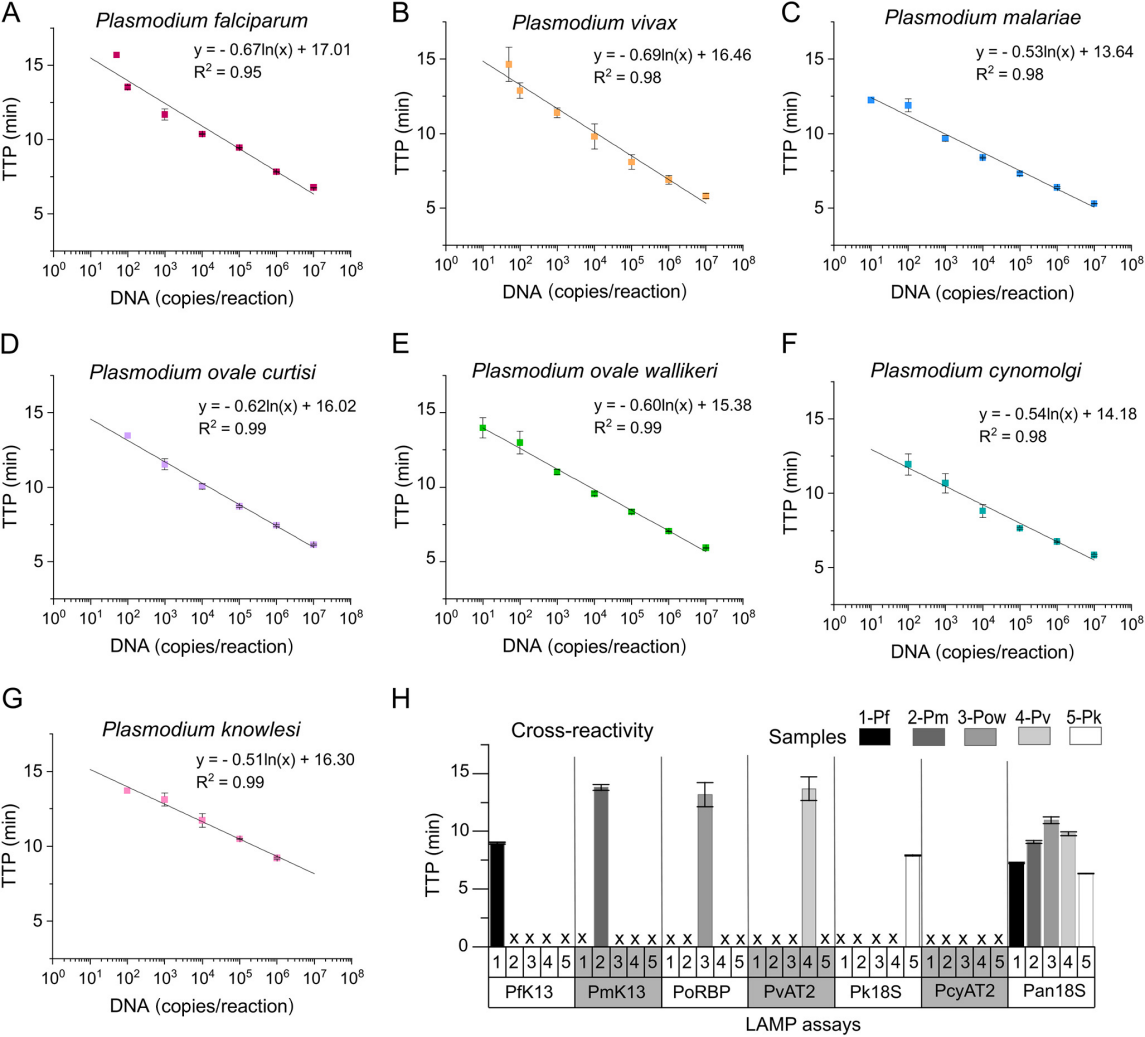
Analytical sensitivity and specificity of species-specific LAMP assays. (A) P. falciparum standard curve (LAMP-PfK13). (B) P. vivax standard curve (LAMP-PvAT2). (C) *P. malariae* standard curve (LAMP-PmK13). (D) *P. ovale curtisi* standard curve (LAMP-PoRBP2), (E) *P. ovale wallikeri* standard curve (LAMP-PoRBP2). (F) P. cynomolgi standard curve (LAMP-PcyAT2). (G) P. knowlesi standard curve (LAMP-Pk18S). The highest concentration available for P. knowlesi was 10^6^ copies per μL. (H) Cross-reactivity of species-specific LAMP assays using clinical isolates (P. falciparum [Pf], *P. malariae* [Pm], *P. ovale wallikeri* [Pow], P. vivax [Pv], and P. knowlesi [Pk]). We have included the TTP values obtained at 50 copies per reaction in A and B. This concentration was not detected in D, F, and G.

Analytical sensitivity was evaluated using synthetic DNA. Standard curves are shown in Fig. 2A to G, with a correlation *R*^2^ of 95 to 99%. TTP values were below 20 min, with a limit of detection of 10 to 100 copies per reaction. Details of the LAMP assays are shown in Table 1, and primer sequences are provided in Table S3. Analytical specificity was assessed *in silico* based on multiple sequence alignment using the MUSCLE algorithm (Fig. S2). Primer sets were highly specific, with no cross-reactivity (primers did not anneal to sequences of other *Plasmodium* species or had a minimum of 4 mismatches per primer). Available clinical isolates (26) of P. falciparum, *P. ovale wallikeri*, *P. malariae*, P. vivax, and P. knowlesi were tested with the species-specific LAMP assays and the LAMP-Pan18S assay. As shown in Fig. 2H, the species-specific LAMP assays amplified only their target isolate, and the LAMP-Pan18S assay detected all.

**TABLE 1:** Summary of the assays used in this work

Assay	Limit of detection (copies/reaction)^*c*^	Gene	Gene abbreviation	TTP (min)	*T_m_* (°C)	Reference
LAMP-PfK13	50	Kelch 13	*K13*	<15^*a*^	78	26
LAMP-PmK13	10	Kelch 13	*K13*	<15^*a*^	79	This study
LAMP-PoRBP2-w	10	Reticulocyte binding protein 2	*RBP2*	<15^*a*^	78	This study
LAMP-PoRBP2-c	100	Reticulocyte binding protein 2	*RBP2*	<15^*a*^	77	This study
LAMP-PvTUBA2	50	Alpha tubulin 2	*TUBA2*	<15^*a*^	87	This study
LAMP-PcyTUBA2	100	Alpha tubulin 2	*TUBA2*	<15^*a*^	85	This study
LAMP-Pk18S	100	18S rRNA	18S	<15^*a*^	83	25
LAMP-Pan18S	50	18S rRNA	18S	<20	85	23
PCR-Pan	1	18S rRNA	18S	40 cycles	NA^*b*^	12
PCR-Pf	1	18S rRNA	18S	40 cycles	NA^*b*^	12

a TTP of lowest detectable DNA concentration. It is recommended to run the assay for 20 min.

b NA, not applicable.

c Reaction volume of 5 μL.

### Performance of LAMP assays in Ghanaian children with symptomatic and asymptomatic P. falciparum parasitemia.

Seventy samples from symptomatic and asymptomatic Ghanaian children were collected in Obom, Ghana. DNA was extracted, and samples were tested with our species-specific LAMP assays, a LAMP assay targeting the human housekeeping gene *ACTB* (LAMP-ACTB) (28), and two TaqMan qPCR assays (12) targeting the 18S rRNA gene of P. falciparum (PCR-Pf) and *Plasmodium* pan-genus (PCR-Pan). Primer sequences are detailed in Table S3. This cohort was also analyzed by microscopy and enzyme-linked immunosorbent assay (ELISA) targeting *Plasmodium* and P. falciparum, respectively.

**FIG 3:**
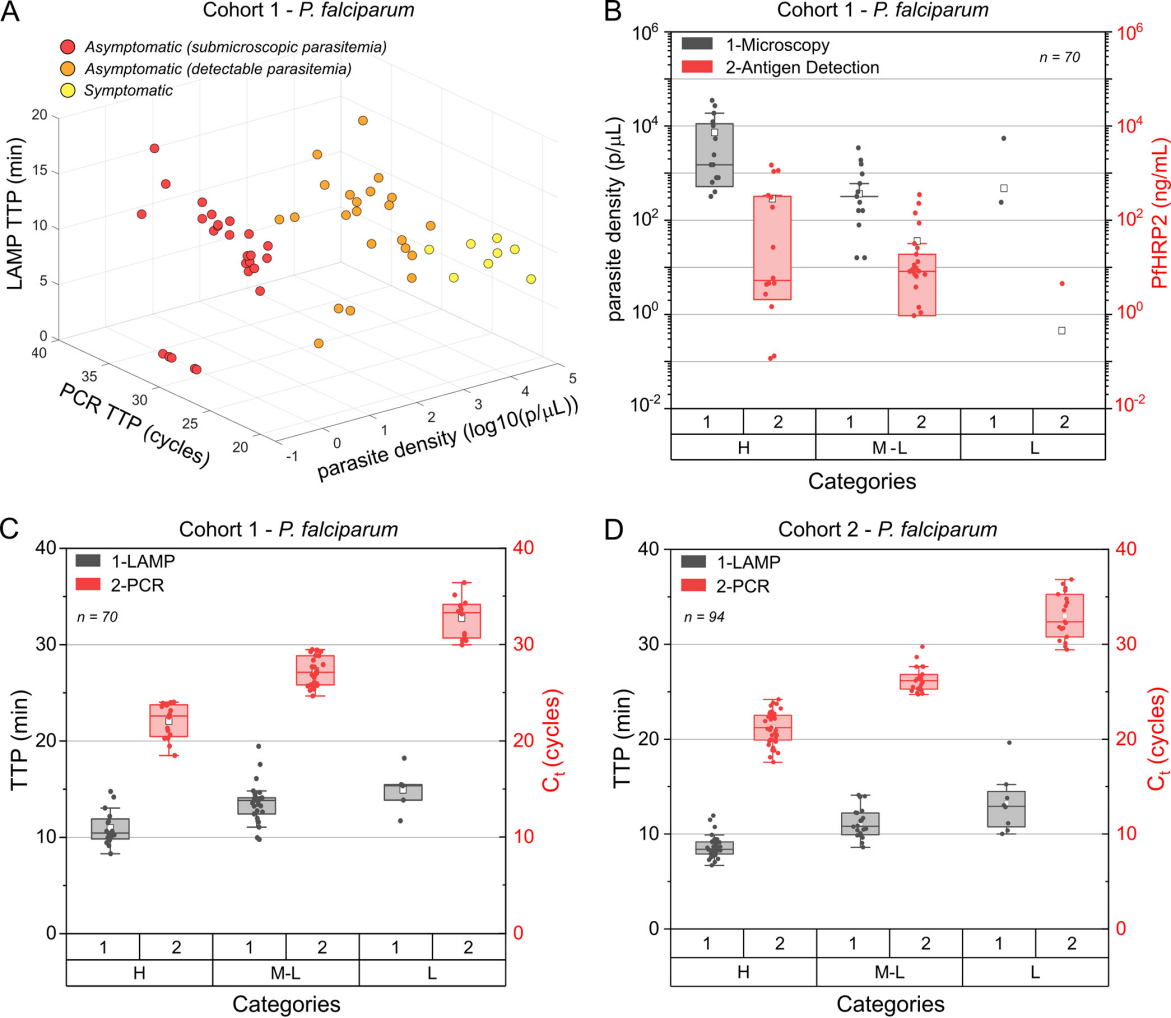
Evaluation of samples from Ghana. (A) Three-dimensional distribution of samples (cohort 1, *N* = 70) stratified according to symptoms, where the *x* axis represents parasite density log_10_ (parasites/μL), the *y* axis represents PCR TTP (cycles), and the *z* axis represents LAMP TTP (min). Dots at “0” indicate that the sample was not detected by the corresponding method. (B) Box and whisker plot showing the distribution of P. falciparum-positive samples detected by microscopy and ELISA across parasitic loads (H, high; M-L, medium-low; L, low). (C) Box and whisker plot showing the distribution of P. falciparum-positive samples detected by PCR-Pf and LAMP-PfK13 in cohort 1 across parasitic loads (H, high; M-L, medium-low; L, low). (D) Box and whisker plot showing the distribution of P. falciparum-positive samples detected by PCR-Pf and LAMP-PfK13 in cohort 2 across parasitic loads (H, high; M-L, medium-low; L, low). Each dot represents a sample; horizontal lines in the boxes indicate medians; lower and upper edges of boxes indicate the interquartile range, and whiskers are less than 1 times the interquartile range.

Based on the symptoms and microscopy results for P. falciparum identification, data were stratified into (i) symptomatic, (ii) asymptomatic with detectable parasitemia, (iii) asymptomatic with submicroscopic parasitemia, and (iv) uninfected. Distribution of the TTPs and parasite density according to this stratification is shown in Fig. 3A, and details are summarized in Table 2. Performance against TaqMan PCR within these 4 groups is detailed as follows. The LAMP-PfK13 assay had 100% (95% confidence interval [95% CI] of 63.1 to 100%) sensitivity (SEN), 100% (95% CI of 78.2 to 100%) specificity (SPE), and 100% (95% CI of 85.2 to 100%) accuracy (ACC) within the symptomatic group. Results within the asymptomatic group with detectable parasitemia were 86.40% (95% CI of 65.1 to 97.1%) SEN, 100% (95% CI of 78.2 to 100%) SPE, and 91.9% (95% CI of 78.1 to 98.3%) ACC. Lastly, 80.77% (95% CI of 60.7 to 93.5%) SEN, 100% (95% CI of 78.2 to 100%) SPE, and 87.8% (95% CI of 73.8 to 95.9%) ACC was achieved within the asymptomatic group with submicroscopic parasitemia, demonstrating the higher SEN of the LAMP assay over microscopy. Results are summarized in Table S4.

**TABLE 2:** Data stratification of cohort 1 using as a reference microscopy and TaqMan PCR assays (*N* = 70)

Category	PCR-Pan *C_T_* (cycles) median (IQR1, IQR3)	PCR-Pf *C_T_* (cycles) median (IQR1, IQR3)	Parasite density (parasite/μL) median (IQR1, IQR3)	Sample (*n*)
Symptomatic	18.36 (17.79, 19.07)	20.68 (20.07, 21.43)	13,943 (4,428.8, 20,814)	8
Asymptomatic^*a*^	24.37 (21.73, 26.28)	27.01 (24.37, 29.07)	1,152.32 (240, 1,410)	21
Asymptomatic^*b*^	26.07 (23.05, 28.22)	28.86 (25.79, 31.12)	NA^*c*^	26
Uninfected	Negative			15
Total				70

a Asymptomatic with detectable parasitemia. IQR, interquartile range.

b Asymptomatic with submicroscopic parasitemia.

c NA, not applicable.

### Correlation of PCR and LAMP in Ghanaian children with symptomatic and asymptomatic P. falciparum parasitemia.

The results obtained with TaqMan PCR were used as a reference to classify the samples into three categories (high, medium-low, and low) based on cycle threshold (*C_T_*) values (Table S5). This three-category division (high, medium-low, and low) matched with the distribution of positives based on the syndromic approach. The ratios of symptomatic to asymptomatic patients were 50% to 50% within the category “high”, 4% to 96% within the category “medium-low” and 0% to 100% within the category “low”.

Using PCR-Pf results as the gold standard, microscopy had an overall 52.7% (95% CI of 39.7 to 67%) SEN, 86.7% (95% CI of 59.5 to 98.3%) SPE, and 60% (95% CI of 48.3 to 72%) ACC. ELISA targeting the P. falciparum histidine-rich protein 2 (*Pf*HRP2) antigen had 67.3% (95% CI of 53.3 to 79.3%) SEN, 86.7% (95% CI of 59.5 to 98.3%) SPE, and 71.4% (95% CI of 59.4 to 81.6%) ACC. Further details are shown in Fig. 3B and are summarized in Tables S5 and S6.

The assay LAMP-PfK13 detected 100% of asymptomatic children in the “high” and “medium-low” categories (*n* = 35), that is, samples presenting more than 80 genomic DNA (gDNA) parasite copies per μL. Furthermore, 83% of all asymptomatic cases (*n* = 47) were detected with this LAMP assay. In this cohort, the LAMP-PfK13 assay had 87.3% (95% CI of 75.5 to 94.7%) SEN, 100% (95% CI of 78.2 to 100%) SPE, and 90% (95% CI of 80.5 to 95.9%) ACC; the LAMP-Pan assay had 90.9% (95% CI of 80.1 to 97%) SEN, 93.3% (95% CI of 68.1 to 99.9%) SPE, and 91.4% (95% CI of 82.3 to 96.8%) ACC. No samples were detected as positive for P. vivax, *P. ovale*, *P. malariae*, P. knowlesi, or P. cynomolgi. Further details are shown in Fig. 3C, Table 3, Table S7, and Fig. S3. The higher SEN and SPE obtained with the LAMP assay are more apparent in the “high” and “medium-low” categories; where microscopy detection achieved 87.5% SPE and 43.8% SEN and antigen detection achieved 93.8% SPE and 71.9% SEN. LAMP-PfK13 achieved 100% SPE and 90.6% SEN, and LAMP-Pan achieved 100% SPE and 96.9% SEN.

**TABLE 3:** Evaluation of samples from Obom (cohort 1) with LAMP-PfK13 and TaqMan PCR-Pf (*N* = 70)

Category^*a*^	PCR-Pf *C_T_* (cycles)	DNA conc. (copies/reaction)^*b*^	Sample (*n*)	TP^*a*^ (*n*)	FN^*a*^ (*n*)	TN^*a*^ (*n*)	FP^*a*^ (*n*)	SEN^*a*^ (%)	SPE^*a*^ (%)	ACC^*a*^ (%)
H	<24	>2.34 ×10^3^	16	16	0			100		
M	24 to 29	2.34 × 10^3^ to 8.03 × 10^1^	27	27	0			100		
L	≥29	≤8.03 ×10^1^	12	5	7			41.7		
N	Not detected		15			15	0			
Total			70	48	7	15	0	87.3	100.0	90.0

a TP, true positives; FN, false negatives; TN, true negatives; FP, false positives; H, high; M-L, medium-low; L, low; U, uninfected; /, Not applicable.

b DNA concentration (copies/reaction) was calculated based on PCR-Pf *C_T_* values using 1 microliter (μL) of sample in a final reaction volume of 10 microliter (μL).

### Evaluation of LAMP assays with a cohort from Cape Coast Ghana.

A second cohort of 94 samples collected from Cape Coast (Ghana) was tested with our species-specific LAMP assays, LAMP-ACTB assay, and PCR-Pf and PCR-Pan TaqMan assays. The LAMP-PfK13 assay had a SEN of 85.4% (95% CI of 75.8 to 92.2%), SPE of 100% (95% CI of 73.5 to 100%), and ACC of 87.2% (95% CI of 78.8 to 93.2%). The LAMP-Pan assay had 80.7% (95% CI of 70.6 to 93.3%) SEN, 100% (95% CI of 71.5 to 100%) SPE, and 83% (95% CI of 73.8 to 90%) ACC. Further details are shown in Fig. 3D, Table 4, Table S8, and Fig. S3. In addition, the SEN values obtained with the LAMP assays in the “high” and “medium-low” categories (parasitic load above 80 gDNA copies per μL) were above 95%; samples belonging to the “low” category with parasitic loads above 40 gDNA copies per μL were also detected with both LAMP assays. The LAMP-PmK13 assay detected 9 samples, all of them also positive by PCR-Pan and PCR-Pf, indicating coinfections and representing 5.5% of the studied subpopulation. No samples were detected as positive for P. vivax, *P. ovale*, P. knowlesi, or P. cynomolgi by any method.

**TABLE 4:** Evaluation of samples from Cape Coast (cohort 2) with LAMP-PfK13 and TaqMan PCR-Pf (*N* = 94)

Category^*a*^	PCR-Pf *C_T_* (cycles)	DNA conc. (copies/μL)^*b*^	Sample (*n*)	TP^*a*^ (*n*)	FN^*a*^ (*n*)	TN^*a*^ (*n*)	FP^*a*^ (*n*)	SEN^*a*^ (%)	SPE^*a*^ (%)	ACC^*a*^ (%)
H	<24	>2.34 × 10^3^	40	40	0			100.0		
M	24 to 29	2.34 × 10^3^ to 8.03 × 10^1^	23	22	1			95.7		
L	≥29	≤8.03 × 10^1^	19	8	11			42.1		
N	Not detected		12			12	0			
Total			94	70	12	12	0	85.4	100	87.2

a TP, true positives; FN, false negatives; TN, true negatives; FP, false positives; H, high; M-L, medium-low; L, low; U, uninfected; /, Not applicable.

b DNA concentration (copies/reaction) was calculated based on PCR-Pf *C_T_* values using 1 microliter (μL) of sample in a final reaction volume of 10 microliter (μL).

All samples included in this study (*n* = 164) were positive by the LAMP-ACTB assay, which was used as an extraction control to verify the human origin of the samples and the quality of extracted DNA. *ACTB* concentration was calculated based on a standard previously built with a positive control. The homogeneous distribution of the *ACTB* concentration across the different categories, including the uninfected samples, indicated optimal quality of the extraction (Fig. S4A). Clinical sensitivity and specificity of the overall study with the LAMP assays (LAMP-PfK13 and LAMP-Pan18S) are summarized in Tables S9 and S10 and Fig. S4B to D.

### Translation into a POC LoC device.

**FIG 4:**
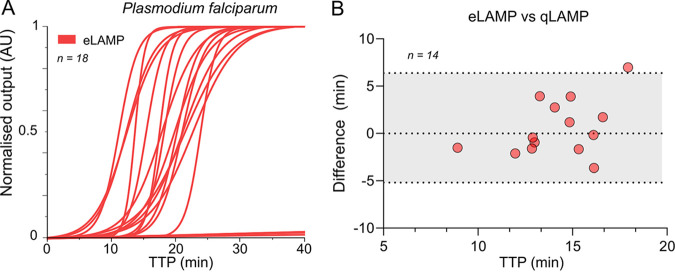
Evaluation of samples in a portable LOC platform in Ghana. (A) Amplification curves of clinical samples (*n* = 18) using our lab-on-chip platform and LAMP assay for P. falciparum detection. (B) Bland-Altman plot showing in the *y* axis the TTP difference between the LOC and real-time instrument and in the *x* axis the mean TTP values with both instruments. Dashed lines represent 95% limits of agreement, being calculated as the mean difference (bias) ± 1.96 times the standard deviation (SD).

A subset of 14 P. falciparum-positive samples and 4 negative samples from the 164 samples were tested on our LoC platform to demonstrate the reliability of a portable device for nucleic acid amplification. In comparison to fluorescence-based instruments, Lacewing relies on electrochemical detection (pH measurements are transduced into electrical signals), which allows label-free real-time monitoring without visual inspection. Furthermore, portability and a digital record of the test leverage its future use as a POC device. Indeed, its transferability and flexibility to accommodate LAMP assays positions this platform as a promising device for digital diagnostics in limited-resource settings and front-line testing (29, 30). As shown in Fig. 4A, amplification curves were obtained with our LoC platform (eLAMP). TTP values were extracted based on the *C_y_* method (26, 31). Samples were tested in parallel in a conventional real-time fluorescence-based instrument (qLAMP). TTP values are summarized in Table S11. Differences between the data obtained with each device are shown in the Bland-Altman plot in Fig. 4B. The distribution of the TTP values was comparable (difference within the 95% limits of agreement) across instruments, indicating the reliability of the proposed device.

## DISCUSSION

The high prevalence of asymptomatic malaria cases (32) and the high rate of misdiagnosis highlight the need of highly sensitive molecular-based methods for diagnosis at the POC. In 2020, malaria cases and deaths increased by 6% and 12%, respectively, compared to in 2019 (1), which was related to disruption in diagnostics, prevention, and treatment during the severe acute respiratory syndrome coronavirus 2 (SARS-CoV-2) pandemic. In 2021, malaria cases increased, and the number of deaths did not decrease significantly. Improvement of current diagnostic strategies, such as microscopy and rapid diagnostic tests (RDTs), to detect subclinical cases and non-P. falciparum infections (33) which are currently underreported (34, 35), could be achieved using molecular digital diagnostics.

To date, there are no commercially available rapid tests based on molecular methods for the specific detection of all non-P. falciparum species. Most distributed tests detect pan-genus or P. falciparum and rely on antigen-specific detection through lateral flow dipsticks (LFDs). Only Eiken chemical (Tokyo, Japan) has deployed a kit (Loopamp malaria pan/Pf/Pv detection kit) for pan-genus, P. falciparum, and P. vivax detection using LAMP. However, laboratory-based instruments are still required to perform the test. Target product profiles for malaria diagnostics should not only meet the REASSURED criteria from the WHO (36) but also focus on the detection of submicroscopic, low-parasite-density-infected populations and species-specific identification for appropriate treatment guidance (37). We describe here the first LAMP assay for the detection of the zoonotic species P. cynomolgi, which may be of clinical relevance at the POC where this species and other zoonotic species (e.g., P. knowlesi) are present. Their use for prospective studies may add valuable information about their epidemiological distribution. In addition, we developed the first LAMP assays for the discrimination between *P. ovale wallikeri* and *P. ovale curtisi*. Due to the common prescription (primaquine) for the dormant liver stage of the *P. ovale* species, we proposed the use of the assays in duplex format. Diagnostics of non-P. falciparum malaria could significantly reduce a reservoir for transmission (38). In the studied cohorts, all the positive cases were P. falciparum, including 9 *P. malariae* coinfections, which corresponded to 5.5% of the population assessed, a percentage that agrees with other studies conducted in Ghana (32, 39).

Detection of low-level parasitemia in symptomatic and asymptomatic carriers is required for epidemiological research and the identification of hot spots for malaria control interventions (37). We demonstrated a SEN of 100% when testing samples from asymptomatic malaria cases with concentrations above 80 gDNA copies per μL and a SEN of 80.77% in asymptomatic cases with submicroscopic parasitemia. These results are higher to ultrasensitive RDTs, such as Alere ultrasensitive malaria Ag P.f (Abbott) RDT (targeting *Pf*HRP2), which achieved a SEN between 55.5% and 73%, as reported in Danwang et al. (40) and Acquah et al. (41). In another study from Yeung et al. (42), this ultrasensitive RDT had a SEN of between 53.8% and 60% compared to qPCR in a low-transmission setting targeting asymptomatic P. falciparum infections. Although deletions in *Pf*HRP2/3 have been widely reported, their prevalence in sub-Saharan Africa remains low (43). We also performed a *Pf*HRP2 ELISA because it is an indicator of total parasite biomass, and its performance reflects the current diagnostic tools available in this region.

Aiming to translate the developed LAMP assays into the POC, we demonstrated as proof of concept the detection of 14 P. falciparum-positive samples with Lacewing, our handheld device. Portable molecular-based digital diagnostics is required to identify asymptomatic malaria cases in limited-resource settings and reduce the burden of this disease. Other platforms, such as the “noninstrumented nucleic acid amplification” (NINA) heater, enable molecular diagnostics to be performed at the POC (44). This platform allows electricity-free, constant, and hermetic incubation to run LAMP reactions. However, this instrument only provides endpoint and, therefore, qualitative results. Our LoC platform performs real-time amplification and provides quantitative data via a dedicated app connected via Bluetooth. This study presents a molecular diagnostic test with the potential to be used with a portable LoC device for P. falciparum and non-P. falciparum digital diagnostics. Although there are some limitations to this study, this introduces a promising technology for malaria control and surveillance. The sample size was small and may not reflect actual prevalence of *Plasmodium* species in Ghana. Also, the developed LAMP assays should be further validated with clinical samples from patients infected with other non-P. falciparum species. A future study could be performed in another setting where multiple species coexist (45). Lastly, to evaluate a truly sample-to-result device at the POC, a low-cost and simple sample preparation module has to be integrated or easily combined with the LoC platform. (28) Future work will address these limitations, providing a reliable molecular-based test for POC malaria diagnostics.

## MATERIALS AND METHODS

### Ethical approval statement.

Collection of samples in Obom between November 2018 and 2019 was approved by the ethical committees of the Noguchi Memorial Institute for Medical Research, University of Ghana (number 024/14-15), and the London School of Hygiene and Tropical Medicine (ID numbers 14322, 15684, and 17257). Written informed consent was obtained from parents or guardians, and assent was appropriately received from the children before they were enrolled in this study (46). Sample collection in Ewim Polyclinic in Cape Coast (Ghana) between the period of July 2018 and December 2019 was approved by the ethics committees of the Ghana Health Service (GHSERC005/12/17), the Noguchi Memorial Institute for Medical Research, University of Ghana (NMIMR-IRB CPN 077/17-18), and the Kintampo Health Research Centre (KHRCIEC/2018-10). All participants and/or guardians of participants gave written informed consent before recruitment (16).

### Clinical samples.

Seventy samples (cohort 1) were collected from children aged 6 to 12 years who were symptomatic, asymptomatic, or uninfected with malaria parasites. The children were recruited between November 2018 and 2019 in Obom, a malaria hyperendemic subdistrict located in Accra, Ghana. Peripheral blood samples (5 mL) were collected into EDTA and PAXgene tubes before any antimalarial treatment and were transported at 4°C to the laboratory for investigation (46). A total of 94 samples (cohort 2) were collected from subjects with suspected malaria. Study participants were recruited at Ewim Polyclinic in Cape Coast (Ghana) between the period of July 2018 and December 2019. A volume of 5 mL of venous blood was collected, and 200 μL of sample was used for DNA extraction with the QIAamp DNA minikit (Qiagen, Manchester, UK) following instructions from the manufacturer. DNA was eluted in a total volume of 100 μL of elution buffer and stored at −20°C.

### Synthetic samples.

Synthetic DNA gBlocks containing the target genes of interest of P. falciparum, *P. ovale curtisi*, *P. ovale wallikeri*, *P. malariae*, P. vivax, and P. cynomolgi were purchased from Thermo Fisher Scientific (USA). Sequence details and reference accession numbers are detailed in Tables S1 and S2 in the supplemental material.

### Microscopy and antigen detection.

Samples in cohort 1 were analyzed by microscopy and enzyme-linked immunosorbent assay (ELISA). Microscopic examination of parasite density was performed by two independent microscopists blinded to each other’s results. Quantification was performed by counting numbers of infected red blood cells relative to white blood cells (WBCs) and assuming a WBC count of 8,000 per μL; at least 500 fields were examined before a smear was classified as negative. The *Pf*HRP2 enzyme immunoassay (malaria antigen CELISA; CeLLabs, Australia; https://www.cellabs.com.au/malaria) was performed on plasma as an indicator of total body parasite load of P. falciparum through the detection of the *Pf*HRP2-specific antigen using plasma samples and following the manufacturer’s instructions (46).

### LAMP primer design.

LAMP assays were designed using the software Primer Explorer version 5.0 (http://primerexplorer.jp/lampv5e/index.html) based on the sequences retrieved from the NCBI GenBank database (https://www.ncbi.nlm.nih.gov/genbank/) and PlasmoDB. Sequences were aligned using the MUSCLE algorithm (47) in Geneious 10.0.5 software (48), and primers were designed to specifically detect the target DNA without interspecies cross-reactivity. All primers were purchased from Integrated DNA Technologies (IDT) and rehydrated in Tris-EDTA (TE) buffer at 100 μM. The LAMP assay specific to the human housekeeping gene *ACTB*, LAMP-ACTB, was used as reported in reference 28. Primer sequences are shown in Table S3.

### LAMP reaction conditions.

LAMP reactions were performed in duplicate with a final volume of 5 μL per reaction. Each reaction contained the following: 0.5 μL of 10× custom isothermal buffer (pH 8.5 to 9), 0.3 μL of MgSO_4_ (100 mM stock), 0.28 μL of dNTPs (25 mM stock), 0.3 μL of bovine serum albumin (BSA; 20 mg/μL stock), 0.8 μL of betaine (5 M stock), 0.5 μL of 10× or 20× LAMP primer mix (with final concentrations per reaction of F3/B3 0.25 or 0.5 μM, LF/LB 1 or 2 μM, and FIP/BIP 2 or 4 μM), 0.13 μL of SYTO9 dye (20 μM stock), 0.13 μL of NaOH (0.2 M stock), 0.03 μL of Bst 2.0 DNA polymerase (120 kU/mL stock), 1 μL of sample, and enough nuclease-free water (Invitrogen) to bring the volume to 5 μL. Reactions were loaded into 96-well plates and were performed at 63°C for 30 min using a LightCycler 96 real-time PCR system (LC96; Roche Diagnostics). One melting cycle was performed at 0.1°C/s from 63°C up to 97°C for validation of the specificity of the amplified products. LAMP-PmK13, LAMP-PoRBP2, and LAMP-ACTB primer mixes were prepared at 20×; the rest of the assays used in this study were prepared at 10×.

### PCR conditions.

TaqMan PCRs were performed in duplicate with a final volume of 10 μL per reaction using the GoTaq Probe qPCR kit from Promega. Each reaction contained the following: 5 μL of 2× GoTaq Probe qPCR master mix, 1 μL of PCR-Pan assay primer/probe mix, 1 μL of PCR-Pf assay primer/probe mix, 1 μL of sample, and enough nuclease-free water to bring the volume to 10 μL. Final concentrations per reaction of the primer mix were forward primer 0.4 μM, reverse primer 0.4 μM, and probe 0.2 μM. Thermal cycling conditions were adapted from Kamau et al. (12) as follows: 1 cycle of 2 min at 95°C, 45 cycles of 15 s at 95°C, and 1 min at 60°C.

### Analytical sensitivity and specificity of the developed LAMP assays.

Analytical sensitivity was evaluated using 10-fold serial dilutions of synthetic DNA ranging from 10^7^ to 10 copies per μL. The highest concentration available for P. knowlesi was 10^6^ copies per μL. In the case that 10^2^ copies per μL was detected but 10 copies per μL was not, a 2-fold dilution to a final concentration of 50 copies per μL was also tested. Dilutions were performed using nuclease-free water. Each condition was run in triplicate using the LC96 real-time instrument. Analytical specificity was performed by testing available clinical isolates (26) of P. falciparum, *P. ovale wallikeri*, *P. malariae*, P. vivax, and P. knowlesi, which were previously screened with the TaqMan PCR-Pan assay, and their calculated concentrations were P. falciparum 1.3 × 10^5^ copies/μL, *P. malariae* 7.7 × 10^2^ copies/μL, *P. ovale wallikeri* 2.2 × 10^3^ copies/μL, P. vivax 6.5 × 10^1^ copies/μL, and P. knowlesi 6.8 × 10^4^ copies/μL.

### LoC platform.

The LoC system consisted of (i) a portable platform powered by a rechargeable battery that contains a Peltier heating module and Bluetooth for data transfer to a smartphone application, (ii) a disposable ion-sensitive field-effect transistor (ISFET) microchip (4,368 sensors, 2 × 4 mm) for pH sensing during nucleic acid amplification, (iii) a microfluidic chamber for containment of the reaction solution (top sealed with PCR tape), which is in contact with the chip through a reference Ag/AgCl electrode, and (iv) a smartphone application (AndoridOS) for real-time data visualization, geotagging, and cloud connectivity for data storage (Amazon web services) (26, 29, 49–51).

### Statistical analysis.

Time-to-positive (TTP) data are presented as mean TTP ± standard deviation. *P* values were calculated by Welch’s unequal variance two sample *t* test or one-way analysis of variance (ANOVA) with a Fisher least significant difference (LSD) means comparison in Origin 2019 (v9.6). A Bland-Altman test was performed in GraphPad using the built-in function for it and calculating the difference versus the average with automatic 95% limits of agreement. A *P* value equal to 0.05 was considered the threshold for statistical significance.

### Study design and sample size.

The samples used in this study were previously tested, and, therefore, samples were known to be positive or negative (16, 46). Consequently, the following calculations (52) were performed to estimate the number of samples required to evaluate the LAMP-PfK13 assay and LAMP-Pan assay,

$$n\;\geq \;\frac{1.96^{2}p(1\;-\;p)}{x^{2}},$$

where *p* is the suspected sensitivity, and *x* is the desired margin of error. We define the true-positive rate (sensitivity) as the proportion of P. falciparum- or *Plasmodium*-positive samples that are correctly detected by the LAMP assays compared to the gold standard TaqMan PCR-Pf and PCR-Pan assays, respectively. We suspected the sensitivity and specificity to be 80% with a desired margin of error of 10%. With these conditions, the number of required samples was 61.47 (rounded up to 62), and we tested a total 164 samples.
