# Supplementary material for: Sensitive Detection of Asymptomatic and Symptomatic Malaria with Seven Novel Parasite-Specific LAMP Assays and Translation for Use at Point-of-Care
Source: Microbiol Spectr. 2023 May 9;11(3):e05222-22. doi: 10.1128/spectrum.05222-22 (PMC10269850; doi:10.1128/spectrum.05222-22)
Supplement: Supplemental file 1 — Supplemental material. Download spectrum.05222-22-s0001.pdf, PDF file, 6.4 MB [file spectrum.05222-22-s0001.pdf]

Supplementary Information
Sensitive detection of asymptomatic and symptomatic malaria with seven novel parasite-specific LAMP assays and translation for use at point-of-care

Kenny Malpartida-Cardenas[1,2], Nicolas Moser[2], Felix Ansah[3], Ivana Pennisi[1], Diana Ahu Prah[3,4], Linda Eva Amoah[3,5], Gordon Awandare[3], Julius Hafalla[3,4], Aubrey Cunnington[1], Jake Baum[6,7], Jesus Rodriguez-Manzano[1,*], and Pantelis Georgiou[2]

[1]Department of Infectious Disease, Faculty of Medicine, Imperial College London, UK

[2]Department of Electrical and Electronic Engineering, Faculty of Engineering, Imperial College London, UK

[3]West African Center for Cell Biology of Infectious Pathogens, Department of Biochemistry, Cell and Molecular Biology, University of Ghana, Ghana

[4]Department of Infection Biology, Faculty of Infectious and Tropical Diseases, London School of Hygiene and Tropical Medicine, London, United Kingdom

[5]Immunology Department, Noguchi Memorial Institute for Medical Research, University of Ghana, Legon, Ghana

[6]Department of Life Sciences, Faculty of Natural Sciences, Imperial College London, UK

[7] School of Biomedical Sciences, University of New South Wales Sydney, Australia

*email: j.rodriguez-manzano@imperial.ac.uk

Table S1. Synthetic DNA sequences (gBlocks).

ID	Sequence (5' to 3')
P. cynomolgi<br>PcyM_0532100	ACTTGGAACCCACGGTGGTAGACGAAGTGCGAACGGGCACCTACCGCCAG<br>CTGTTTCACCCAGAGCAGTTAATATCAGGAAAGGAAGACGCAGCAAATAA<br>TTTCGCAAGAGGACACTACACAATTGGAAAGGAAATAGTTGACATATGCT<br>TAGATCGAGTGAGAAAGTTAGCTGACAATTGCACAGGGCTGCAGGGTTTT<br>CTCATGTTTAATGCAGTGGGTGGAGGAACGGGAAGTGGCCTGGGGTGTCT<br>TCTGTTGGAAAGGCTAGCCATAGATTATGGAAAGAAATCCAAATTAAATT<br>TCTGTTCCTGGCCCTCTCCCCAAGTATCCACAGCTGTGGTGGAACCATAC<br>AACTCAGTTTTATCGACCCACTCTCTACTAGAACACACAGATGTAGCGAT<br>CATGTTAGATAATGAAGCTATATACGACATATGTAAAAAAAATTTGGACA<br>TAGAAAGGCCTACTTATACAAACCTAAACAGACTAATAGCTCAGGTCATT<br>TCTTCCCTAACGGCTTCTCTACGATTTGATGGTGCCCTAAATGTGGATGT<br>TACGGAATTTCAAACGAACTTAGTACCTTACCCACGTATCCATTTTATGT<br>TATCATCGTACGCCCCTATCATTAGTGCAGAGAAGGCA
P. vivax<br>PVP01_0530800	GTGCCTCGCTGCGTGTTCGTGGACTTGGAACCCACGGTGGTGGACGAAGT<br>GCGAACGGGGACCTACCGCCAGCTGTTTCACCCAGAGCAGCTAATCTCCG<br>GCAAGGAAGACGCAGCAAACAACTTCGCCAGGGGGCACTACACAATTGGG<br>AAGGAAATCGTAGACGTCTGTTTAGATCGAGTGAGAAAGTTGGCGGACAA<br>TTGCACAGGGCTTCAAGGCTTTCTCATGTTCAATGCCGTGGGTGGAGGAA<br>CAGGAAGTGGTCTGGGATGTCTTTTGTTAGAAAGGCTAGCCATAGATTAC<br>GGGAAGAAATCGAAGTTGAATTTTTGCTCCTGGCCTTCCCCTCAAGTCTC<br>CACAGCTGTGGTGGAACCATATAACTCGGTTCTGTCTACCCACTCTCTTC<br>TAGAACACACAGATGTAGCGATTATGTTAGATAATGAAGCTATCTACGAC<br>ATATGTAAAAAGAATTTAGATATTGAAAGGCCGACCTATACAAACCTAAA<br>CAGATTAATAGCTCAGGTCATTTCCTCGTTGACGGCTTCTCTCCGTTTTG<br>ATGGTGCCTTAAATGTAGACGTTACTGAATTTCAAACGAACTTAGTGCCC<br>TACCCACGTATCCATTTCATGCTGTCGTCTTACGCCCCCATCATTAGCGC<br>TGAGAAGGCATATCACGAGCAGCTGTCCGTTTCGGAAATTACAAACTCTG<br>CATTTGAGCCTGCTTCTATGATGGCCAAGTGTGACCCTCGTCATGGCAAA<br>TACATGGCTTGCTGCCTAATGTACAGGGGTGATGTCGTGCCGAAGGATGT<br>CAATGCTGCTGTTGCGACTATTAAGACTAAGAGGTCCATTCAGTTTGTTG<br>ACTGGTGCCCAACTGGATTCAAATGTGGTATTAACTACCAACCACCGACG<br>GTTGTCCCTGGGGGAGATTTGGCCAAAGTGATGAGAGCCGTCTGCATGAT<br>AAGTAACTCCACTGCAATTGCTGAAGTGTTTTCTCGAATGGACCAGAAAT<br>TTGACCTCATGTATGCTAAGAGGGCGTTCGTACACTGGTACGTTGGAGAA<br>GGTATGGAGGAGGGGGAGTTCAGTGAAGCCAGGGAGGACTTGGCCGCTTT<br>GGAAAAGGACTACGAAGAGGTCGGCATCGAGACCAACGAGGGAGAAGGCG<br>AAGACGAGGGCTACGAATGA
P. falciparum<br>PF3D7_1343700.1	CGATTTGATGAAGAAAGATTAAGATTTTTACAAGAAATCGATAAAATTAA<br>ATCATGTAACCAGAGATAAACAAGGAAGAATATTCTTAGATAGGGATAGT<br>GAGTTATTTAGAATTATACTTAACTTCTTAAGAAATCCGTTAACTATACC<br>CATACCAAAAGATTTAAGTGAAAGTGAAGCCTTGTTGAAAGAAGCAGAAT<br>TTTATGGTATTAAATTTTTACCATTCCCATTAGTATTTTGTATAGGTGGA<br>TTTGATGGTGTAGAATATTTAAATTCGATGGAATTATTAGATATTAGTCA<br>ACAATGCTGGCGTATGTGTACACCTATGTCTACCAAAAAAGCTTATTTTG<br>GAAGTGCTGTATTGAATAATTTCTTATACGTTTTTGGTGGTAATAACTAT<br>GATTATAAGGCTTTATTTGAAACTGAGGTGTATGATCGTTTAAGAGATGT<br>ATGGTATGTTTCAAGTAATTTAAATATACCTAGAAGAAATAATTGTGGTG<br>TTACGTCAAATGGTAGAATTTATTGTATTGGGGGATATGATGGCTCTTCT<br>ATTATACCGAATGTAGAAGCATATGATCATCGTATGAAAGCATGGGTAGA<br>GGTGGCACCTTTGAATACCCCTAGATCATCAGCTATGTGTGTTGCTTTTG<br>ATAATAAAATTTATGTCATTGGTGGAACTAATGGTGAGAGATTAAATTCT<br>ATTGAAGTATATGAAGAAAAAATGAATAAATGGGAACAATTTCCATATGC<br>CTTATTAGAAGCTAGAAGTTCAGGAGCAGCTTTTAATTACCTTAATCAAA<br>TATATGTTGTTGGAGGTATTGATAATGAACATAACATATTAGATTCCGTT<br>GAACAATATCAACCATTTAATAAAAGATGGCAATTTCTAAATGGTGTACC<br>AGAGAAAAAAATGAATTTTGGAGCTGCCACATTGTCAGATTCTTATATAA<br>TTACAGGAGGAGAAAATGGCGAAGTTCTAAATTCATGTCATTTCTTTTCA<br>CCAGATACAAATGAATGGCAGCTTGGCCCATCTTTATTAGTTCCCAGATT<br>TGGTCACTCCGTTTTAATAGCAAATATATAA

Table S1. Synthetic DNA sequences (gBlocks).

ID	Sequence (5' to 3')
P. malariae<br>PmUG01_12021200.1	ATGGAAGGAGAAAAAATAAAATCCAACAGTATTTCAAATTTTTCTGTCAC<br>GTATGATAGAGAATCCGGTGTTAACAGCAACAGCGATGACAGAAGTGAAA<br>GTAGTAGTGAAAATGAGTCTAATTCATTTATGAATATGACAAGCGATAAA<br>AATGAAAAAACAGAAAATAATAGTTTTGCATTAAATAATAGCAGTTTTGT<br>AAACATGAAAGATAGTTTATTAGAGTCGATAGATTTGAGTGTATTAGATT<br>CAAACTTTGATACGAAAAAAGATTTTTTACCAAGTAATTTTTCAAAAAAC<br>CCCAATAATTTATCAAAAGAAAATATTAGTGGCTGGCCCCCGTCCAAATT<br>TTTAAATAAAAGTGATTCGATGTTTATGTCGAAGAGTAAAGACATGAACT<br>TAACGGACGCAAGTAATAATAATGTGAACATATCCGTAAAAAATAACACG<br>AAAAAAGAAATTTTTATGGATGCCGCAACAGCATCTTTAAATGCGAACGA<br>GGAAAATGCAATGAATAATTTAAAAAAGTTTACGAATACAAAGGCCCTCC<br>CCCATGATACATATGAAAAGAAAATAATCGAAACCGAGTTAAGTGATTCC<br>AGTGATTTTGAAAACATGGTAGGTGATTTAAGAATCACTTTTATAAATTG<br>GTTAAAGAAGACACAGATGAATTTTATGAAACTATGATAGATATTAACGT<br>TGGGGGAGCTATTTTTGAAACATCCAGACATACGTTAACTCAACAGAAAG<br>ATTCTTTTATAGAAAAACTATTAAGTGGTAGATATCATGTAACAAGAGAT<br>AAACAGGGTAGAATATTTTTAGATCGTGATAGTGAATTATTTAGAATTAT<br>ATTAAACTTCTTAAGAAATCCTTTAACTGTTCCAATACCAAAAGATTTGA<br>GTGAAAGTGAAGCATTATTAAAAGAGGCAGAATTTTATGGTTTAAACAAT<br>TTCTTGTATGTATTCGGAGGAAATAATTATGATTATAAAGCATTATTTGA<br>AACAGAAGTGTATGATCGTTTGAGAGATACATGGTTTGTTTCAAGTAATT<br>TAAATATACCAAGAAGAAATAATTGTGGTGTTACATCCAATGGTAGAATT<br>TATTGTATTGGTGGTTATGATGGTTCCTCCATTATACCAAATGTTGAAGC<br>TTATGATCATCGGATGAAAGCATGGGTAGAAATTGCACCTTTAAATACTC<br>CAAGATCTTCATCCA
P.o. curtisi<br>GU813971.1	ATGTTGGAAACAACTGACACCTTAGATTATATTGATGGTACAGATAACGAGAA<br>AAATATAATTTCCCAGTTAAAACCAGACTACTCTTATGTATATTATTT<br>CAACGAAATTAAACGCTATGCAGAATATCACAAAGAAATATCTCCCAAATAT<br>GAAAGTATATATAATTCTAGCATCAAAACCTTAAAAGAATACATAGAAAATG<br>CAGTCGATACATGTAAACCTAAGAAAAATGAAATGATTGCTTTAACAAAAAT<br>TTTAGAAGATCCTGAAAAAATTAAGGGACTTGAAGGACATTATGAAGGAAAA<br>CTTCATGCATACAAAACATATATGAAGGAATATCAAAACTGTTTAATAAATA<br>AAAGCAATAAAACTATGCCACAGATAAGAAGTCTCAAGTACGATATTAATGA<br>ATTGCTAAGCGATATCAATTGCAAACAAAAGTGCTCCAATGAAACTTATAAT<br>AATATGATTAAGATATATTTACTAGAATTTAACAATGTACCGTACGAAACAC<br>ACATCCAATATATAAAGAACTTTAAAAGTTCTTTGGACTTTGGAAATCATTT<br>AATAAATAGGGTACAAAGAGAATTAGGCAAAAATAATATTATAGACTCAACGA<br>AATTTTTACAGGAAGAAATAAAATATATTATAGAAAGATTTTATACCCATTT<br>AGATAAAGTCAAATATGGAATGGATTATATAAAGGATCTTTCCAAGAAAGAAG<br>TCCTAAATGAAGTTACCATAGATGTCCTTAAAAATAATTATATTACGCTACCT<br>TACTATTACGCCC
P.o. wallikeri<br>GU813972.1	ATGTTGGAAACAACTGACACCTTAGATTATATTGATGGCACAGATAACGAGAA<br>AAATATAATTTCCCAGTTAAAACCAGACTACGCTTATGTATATTATTTCAACG<br>AAATTAAACGCTATACAGAATATCACAAAGAAATATCTTCCAAATATGAAAGT<br>ATATATAATTCTAGCATCAAAACCTTAAAAGAAGACATAGAAAATGCAGTCGA<br>TACATGTAAACCTAAGAAAAATGAAATGATTGCTTTGACAAAAATTTTAGAAG<br>ATCCTGAAAAAATTAAGGGACTTGAAGGGCATTATGAAGGAAAATTTCATGCAT<br>ACAGAACATATATGAAGGAATATCAAAACTGTTTAATAAATAAAAGCAATAAAA<br>CGATGCCACAGATAAGAAGTCTCAAGTACGATATTAATGAATTGCTAAGCGATA<br>TCATTTGCAAACAAAAGTGCTCCAATGAAACCTATAATAATATGATTAAGATAT<br>ATTTACTAGAATTTAACAATGTACCGTACGAAACGCACATCCAATATATAAAGA<br>ACATTAAAAGTTCTTTGGACTTTGGGAGTCATTTAATAAATAGGGCACAAAGAG<br>AATTAGGCAAAAGTAATATTATAGACTCAACGAAATTTGTACAGGAAGAAATAA<br>AATATATTATAGAAAGATTTTATACCCATTTAGATAAAGTCAAATATGGAATGG<br>ATTATATAAAGGATCTTTCCAAGAAAGAAGTCCTAAATGAAGTGACCTTAGATG<br>TCCTTAAAAATAATTATATTACGCTACCTTACTATTACGCCC

Table S2. Reference sequences accession numbers.

Kelch 13 (K13)	Alpha-Tubulin (TUBA2)	Alpha-Tubulin (TUBA2)	Reticulocyte Binding Protein 2 (RBP2)
PmUG01_12021200.1	PcyM_0532100	PADL01_0421000	GU813971.1
PocGH01_12019400.1	PCYB_053570	PfML01_040028800	JF894427.1
PF3D7_1343700	PVP01_0530800	PfSN01_040026800	KU510234.1
PKNOH_S09541100t35_1	PVX_090155	PfGA01_040026700	JF894428.1
PKNH_1257700.1	XM_002258237.1	PfGA01_040026700	GU813972.1
PVX_083080.1	PocGH01_05032600	PRELSG_0516800	JF894422.1
PcyM_1212800t36_1	PmUG01_05038900t	YYE_03958	JF894425.1
PVP01_1211100.1	PF3D7_0422300	PRG01_0426900	JF894426.1
PCHAS_1361300.1	PfTG01_040028100	PYYM_0523200	MG251661.1
PCYB_122000	PCOAH_00012520	PY17X_0524100	JF894423.1
KT792970.1	AK88_05372	PY04063	
KT792969.1	PKNH_0514200	PGAL8A_00192500	
PPRFG01_1345600.1	PKNOH_S04366800	PocGH01_05032600	
PfGA01_130049800t41_1	PCHAS_0522900	PmUG01_05038900	
PfGB4_130049600t41_1	YYG_03587	PfKH01_090008100	
PfHB3_130050000t41_1	PRCDC_0419500	PfCD01_090008100	
PfTG01_130049400t41_1	PBLACG01_0420400	PfSD01_090008800	
PfSN01_130046700t41_1	PGABG01_0419300	PfML01_090008200	
PfKH02_130046600t41_1	PfHB3_040025700	PfTG01_090008100	
Pf7G8_130048100t41_1	PBILCG01_0415500	PfKE01_090008100	
PfCD01_130049300t41_1	PGSY75_0422300	PfIT_090008400	
PfGN01_130050400t41_1	PfKE01_040028800	PfDd2_090009000	
PfKE01_130049300t41_1	PF3D7_0422300	PfKH02_090008500	
PfML01_130047600t41_1	PfIT_040026800	PfGA01_090008100	
PfSD01_130050400t41_1	PBANKA_0522700	PfGB4_090008700	
PfDd2_130049500t41_1	PfTG01_040028100	PfSN01_090008400	
PfIT_130049000t41_1	PfDd2_040027300	PfHB3_090008400	
PfKH01_130047700t41_1	Pf7G8_040026600	PfGN01_090008600	
	PfGN01_040027400	Pf7G8_090008600	
	PfKH01_040028700	PVX_098630	
	PfSD01_070037900	PY01155	
	PfKH02_040027700	AK88_00920	
	PfGB4_040027000	PCOAH_00018900	
	PPRFG01_0420000	PKNOH_S06402900	
	PfCD01_040027500	PKNH_0701400	
	YYG_04031	PCYB_071150	

Table S3. Primer sequences of LAMP assays and PCR assays targeting human-malaria.

Assay	Primer	Sequence (5' to 3')	Author	Reference
LAMP-ACTB	F3	GCGCGGCTACAGCTTCA	Malpartida-Cardenas et al.	[29] LAMP-ACTB
B3	GGAAGAGTGCCTCAGGGC			
LAMP-ACTB	LF	AGCTTCTCCTTAATGTCACGC		
LAMP-ACTB	LB	CGAGCTGCCTGACGG		
LAMP-ACTB	FIP	TTGCTCGAAGTCCAGGGCGACGAGCGGGAAATCGTGC		
LAMP-ACTB	BIP	TGCTTCCAGCTCCTCCCTGATTGCCAATGGTGATGACCT		
LAMP-PmK13	F3	AAATAAAATCCAACAGTATTTCAAATTTT	This study	This study
LAMP-PmK13	B3	TCGTATCAAAGTTTGAATCTAATAC		
LAMP-PmK13	LF	GCTGTTGCTGTTAACACC		
LAMP-PmK13	LB	AATAGCAGTTTTGTAAACATGAAAGAT		
LAMP-PmK13	FIP	TTCACTACTACTTTCACTTCTGTCATC-TCTGTCACGTATGATAGAGAATC		
LAMP-PmK13	BIP	CATTTATGAATATGACAAGCGATAAAAATGAAA-ACTCAAATCTATCGACTCTAATAAACT		
LAMP-PoRBP2	F3-c	CCCAGTTAAAACCAGACTACG	This study	This study
LAMP-PoRBP2	B3-c	CTGTATGCATGAAATTTTCCTTCA		
LAMP-PoRBP2	LF-c	ACTTTCATATTTGGGAGATATTTCTTTGTG		
LAMP-PoRBP2	LB-c	TGCTTTAACAAAAATTTTAGAAGATCCT		
LAMP-PoRBP2	FIP-c	TGTATTCTTTTAAGGTTTTGATGCTAGAATT-ACGAAATTAAACGCTATGCAGAATA		
LAMP-PoRBP2	BIP-c	TGCAGTCGATACATGTAAACCTAAGAAA-GTCCTTCAAGTCCCTTAATTTTTTC		
LAMP-PoRBP2	F3-w	TTCCCAGTTAAAACCAGACTAC		
LAMP-PoRBP2	B3-w	TGTATGCATGAAATTTTCCTTC		
LAMP-PoRBP2	LF-w	ACTTTCATATTTGGAAGATATTTCTTTGTG		
LAMP-PoRBP2	LB-w	GCTTTGACAAAAATTTTAGAAGATCCT		
LAMP-PoRBP2	FIP-w	TGTCTTCTTTTAAGGTTTTGATGCTAGAA-CAACGAAATTAAACGCTATACAGAATA		
LAMP-PoRBP2	BIP-w	TGCAGTCGATACATGTAAACCTAAGAAA-CCCTTCAAGTCCCTTAATTTTTTC		
LAMP-PvTUBA2	F3	GTGGACGAAGTGCGAACG	This study	This study
LAMP-PvTUBA2	B3	CTTTCTAACAAAAGACATCCCAGAC		
LAMP-PvTUBA2	LF	GCCGGAGATTAGCTGCTCT		
LAMP-PvTUBA2	LB	AATTGCACAGGGCTTCAAGG		
LAMP-PvTUBA2	FIP	CCCCCTGGCGAAGTTGT-TACCGCCAGCTGTTTCAC		
LAMP-PvTUBA2	BIP	TCGTAGACGTCTGTTTAGATCGAGT-ACCCACGGCATTGAACATG		

Table S3. Primer sequences of LAMP assays and PCR assays targeting human-malaria.

Assay	Primer	Sequence (5' to 3')	Author	Reference
LAMP-PfK13	F3	GGAGCAGCTTTTAATTACCTT	Malpartida-Cardenas et al.	[31]
LAMP-PfK13	B3	ATGACATGAATTTAGAACTTCGCC		
LAMP-PfK13	LF	AATATGTTATGTTCATTATCAA		
LAMP-PfK13	LB	GAGAAAAAAATGAATTTTGGAGCT		
LAMP-PfK13	FIP	TGGTTGATATTGTTCAACGGAATCT-ATCAAATATATGTTGTTGGAGGT		
LAMP-PfK13	BIP	TGGCAATTTCTA AATGGTGTACCA-ATAAGAATCTGACAATGTGGC		
LAMP-PcyTUBA2	F3	CAATTGGAAAGGAAATAGTTGACAT	This study	This study
LAMP-PcyTUBA2	B3	GGTCGATAAAACTGAGTTGTATGG		
LAMP-PcyTUBA2	LF	GCCCTGTGCAATTGTCAG		
LAMP-PcyTUBA2	LB	GAAATCCAAATTAAATTTCTGTTCCTGG		
LAMP-PcyTUBA2	FIP	GCATTAAACATGAGAAAACCCTGCA-ATGCTTAGATCGAGTGAGAAAGTTA		
LAMP-PcyTUBA2	BIP	CTGTTGGAAAGGCTAGCCATAGATTATGGA-GTGGATACTTGGGGAGAGG		
LAMP-Pan18s	F3	GTATCAATCGAGTTTCTGACC	Han et al.	[28]
LAMP-Pan18s	B3	CTTGTCACTACCTCTCTTCT		
LAMP-Pan18s	LF	CGTCATAGCCATGTTAGGCC		
LAMP-Pan18s	LB	AGCTACCACATCTAAGGAAGGCAG		
LAMP-Pan18s	FIP	TCGAACTCTAATTCCCCGTTACCTATCAGCTTTTGATGTTAGGGT		
LAMP-Pan18s	BIP	CGGAGAGGGAGCCTGAGAAATAGAATTGGGTAATTTACGCG		
LAMP-Pk18s	F3	CCATCTATTTCTTTTTTGCGTATG	Lau et al.	[27]
LAMP-Pk18s	B3	CAGTGGAGGAAAAGTACGAA		
LAMP-Pk18s	LF	TAGACACACATCGTT		
LAMP-Pk18s	LB	GCACGCGTGCTACACT		
LAMP-Pk18s	FIP	GTTGTTGCCTTAAACTTCCTTGTGTTCTTGATTGTAAAGCTTCTTAGAGG		
LAMP-Pk18s	BIP	TGATGTCCTTAGATGAACTAGGCTTTGCAAGCAGCTAAAATCGT		
PCR-Pan	F	GCTCTTTCTTGATTTCTTGGATG	Kamau et al.	[32]
PCR-Pan	R	AGCAGGTTAAGATCTCGTTCG		
PCR-Pan	P	/5Cy5/ATGGCCGTT/TAO/TTTAGTTCGTG/3IAbRQSp/		
PCR-Pf	F	ATTGCTTTTGAGAGGTTTTGTTACTTT	Kamau et al.	[32]
PCR-Pf	R	GCTGTAGTATTCAAACACAATGAACTCAA		
PCR-Pf	P	/56-FAM/CATAACAGACGGGTAGTCAT/3BHQ_1/		

Table S4. Results obtained with LAMP-PfK13 and LAMP-Pan18S with cohort 1 (N = 70) according to syndromic stratification.

		**LAMP-PfK13**						**LAMP-Pan18S**					
**Symptoms**	**Sample (n)**	**TP (n)**	**TN (n)**	**FP (n)**	**FN (n)**	**SEN**	**SPE**	**TP (n)**	**TN (n)**	**FP (n)**	**FN (n)**	**SEN**	**SPE**
**Symptomatic**	8	8	/	/	0	100.00		8	/	/	0	100.00	
**Asymptomatic**	21	19	/	/	2	90.48		20	/	/	1	95.24	
**Asymptomatic**	26	21	/	/	5	80.77		23	/	/	3	88.46	
**Uninfected**	15	/	15	/	/			/	15	/	/		
Total	70	48	15	0	7	87.27	100	51	15	0	4	92.73	100

[a] Asymptomatic with detectable parasitemia
[b] Asymptomatic with submicroscopis parasitemia

Table S5. Results obtained from microscopy examination and TaqMan PCR-Pf (n = 70).

**Cat.**	**PCR-Pf Ct (cycles)**	**DNA Conc. (copies/reaction)***	**Sample (n)**	**TP (n)**	**FN (n)**	**TN (n)**	**FP (n)**	**SEN(%)**	**SPE(%)**	**ACC(%)**
**H**	< 24	$> 2.34 \times 10^3$	16	14	2	/	/	87.5	/	/
**M-L**	[24 - 29)	$[2.34 \times 10^3 - 8.03 \times 10^1)$	27	13	14	/	/	48.1	/	/
**L**	≥ 29	$\leq 8.03 \times 10^1$	12	2	10	/	/	16.7	/	
**U**	negative		15	/	/	13	2	/	/	/
TOTAL			70	29	26	13	2	52.7	86.7	60

*DNA concentration (copies/reaction) was calculated based on PCR-Pf Ct values using 1 µL of sample in a final reaction volume of 10 µL.
"TP" true positives; "FN" false negatives; "TN" true negatives; "FP" false positives. "H" high; "M-L" medium-low; "L" low; "U" uninfected.

Table S6. Results obtained using the RDT Malaria Antigen (HRP-2) from CELISA and TaqMan PCR-Pf (n = 70).

**Cat.**	**PCR-Pf Ct (cycles)**	**DNA Conc. (copies/reaction)***	**Sample (n)**	**TP (n)**	**FN (n)**	**TN (n)**	**FP (n)**	**SEN(%)**	**SPE(%)**	**ACC(%)**
**H**	< 24	$> 2.34 \times 10^3$	16	15	1	/	/	93.8	/	/
**M-L**	[24 - 29)	$[2.34 \times 10^3 - 8.03 \times 10^1)$	27	21	6	/	/	77.8	/	/
**L**	≥ 29	$\leq 8.03 \times 10^1$	12	1	11	/	/	8.3	/	
**U**	negative		15	/	/	13	2	/	/	/
TOTAL			70	37	18	13	2	67.3	86.7	71.4

*DNA concentration (copies/reaction) was calculated based on PCR-Pf Ct values using 1 µL of sample in a final reaction volume of 10 µL.

"TP" true positives; "FN" false negatives; "TN" true negatives; "FP" false positives. "H" high; "M-L" medium-low; "L" low; "U" uninfected.

Table S7.Evaluation of samples from Obom with LAMP-Pan18S and TaqMan PCR-Pan (n = 70).

**Cat.**	**PCR-Pan Ct (cycles)**	**DNA Conc. (copies/reaction)***	**Sample (n)**	**TP (n)**	**FN (n)**	**TN (n)**	**FP (n)**	**SEN(%)**	**SPE(%)**	**ACC(%)**
**H**	< 22	$> 2.34 \times 10^3$	16	16	0	/	/	100	/	/
**M-L**	[22 - 27)	$[2.34 \times 10^3$ – $8.03 \times 10^1)$	27	27	0	/	/	100	/	/
**L**	≥ 27	$\leq 8.03 \times 10^1$	12	7	5	/	/	58.3	/	
**U**	negative		15	/	/	14	1	/	/	/
TOTAL			70	50	5	14	1	90.9	93.3	91.4

*DNA concentration (copies/reaction) was calculated based on PCR-Pan Ct values using 1 µL of sample in a final reaction volume of 10 µL.
"TP" true positives; "FN" false negatives; "TN" true negatives; "FP" false positives. "H" high; "M-L" medium-low; "L" low; "U" uninfected

Table S8. Evaluation of samples from Cape Coast with LAMP-Pan18S and TaqMan PCR-Pan (n = 94).

**Cat.**	**PCR-Pan Ct (cycles)**	**DNA Conc. (copies/reaction)***	**Sample (n)**	**TP (n)**	**FN (n)**	**TN (n)**	**FP (n)**	**SEN(%)**	**SPE(%)**	**ACC(%)**
**H**	< 22	$> 2.34 \times 10^3$	40	39	1	/	/	97.5	/	/
**M-L**	[22 - 27)	$[2.34 \times 10^3$ – $8.03 \times 10^1)$	23	22	1	/	/	95.7	/	/
**L**	≥ 27	$\leq 8.03 \times 10^1$	20	6	14	/	/	30	/	
**U**	negative		11	/	/	11	0	/	/	/
TOTAL			94	67	16	11	0	80.7	100	83

*DNA concentration (copies/reaction) was calculated based on PCR-Pan Ct values using 1 µL of sample in a final reaction volume of 10 µL.
"TP" true positives; "FN" false negatives; "TN" true negatives; "FP" false positives. "H" high; "M-L" medium-low; "L" low; "U" uninfected

Table S9. Results obtained with LAMP-PfK13 and TaqMan PCR-Pf (n = 164).

**Cat.**	**PCR-Pf Ct (cycles)**	**DNA Conc. (copies/reaction)***	**Sample (n)**	**TP (n)**	**FN (n)**	**TN (n)**	**FP (n)**	**SEN(%)**	**SPE(%)**	**ACC(%)**
**H**	< 24	$> 2.34 \times 10^3$	56	56	0	/	/	100	/	/
**M-L**	[24 - 29)	$[2.34 \times 10^3 - 8.03 \times 10^1)$	50	49	1	/	/	98	/	/
**L**	≥ 29	$\leq 8.03 \times 10^1$	31	13	18	/	/	42	/	
**U**	negative		27	/	/	27	0	/	/	/
TOTAL			164	118	19	27	0	86.1	100	88.4

*DNA concentration (copies/reaction) was calculated based on PCR-Pf Ct values using 1 µL of sample in a final reaction volume of 10 µL.
"TP" true positives; "FN" false negatives; "TN" true negatives; "FP" false positives. "H" high; "M-L" medium-low; "L" low; "U" uninfected.

Table S10. Results obtained with LAMP-Pan18S and TaqMan PCR-Pan (n = 164).

**Cat.**	**PCR-Pan Ct (cycles)**	**DNA Conc. (copies/reaction)***	**Sample (n)**	**TP (n)**	**FN (n)**	**TN (n)**	**FP (n)**	**SEN(%)**	**SPE(%)**	**ACC(%)**
**H**	< 22	$> 2.34 \times 10^3$	56	55	1	/	/	98.2	/	/
**M-L**	[22 - 27)	$[2.34 \times 10^3 - 8.03 \times 10^1)$	50	49	1	/	/	98	/	/
**L**	≥ 27	$\leq 8.03 \times 10^1$	32	13	19	/	/	40.6	/	
**U**	negative		26	/	/	25	1	/	/	/
TOTAL			164	117	21	25	1	84.8	96.2	86.6

*DNA concentration (copies/reaction) was calculated based on PCR-Pan Ct values using 1 µL of sample in a final reaction volume of 10 µL.
"TP" true positives; "FN" false negatives; "TN" true negatives; "FP" false positives. "H" high; "M-L" medium-low; "L" low; "U" uninfected

Table S11.TTP values of the samples tested on the Lab-on-Chip platform.

**Sample** ID	**Category**	**Conc. (copies/reaction)**	**Ct_LW** (min)	**Cy_LW** (min)	**TTP_LC96** (min)	**TTP ± STD** (min)	**PCR-Pf** Mean ± STD (cycles)
1	H	5.46E+04	11.33	10.91	13.02	8.25 ± 0.10	19.38 ± 0.04
2	H	1.94E+04	16.12	15.43	12.68	8.25 ± 0.07	20.93 ± 0.10
3	M-L	5.33E+02	13.58	12.68	13.10	10.08 ± 0.45	26.34 ± 0.16
4	H	6.81E+03	17.02	15.23	11.29	7.9 ± 0.10	22.51 ± 0.09
5	H	7.20E+03	16.17	14.49	16.15	8.36 ± 0.12	22.42 ± 0.07
6	L	5.45E+01	18.58	17.46	15.73	10.37 ± 0.07	29.77 ± 0.12
7	M-L	1.57E+03	22.22	21.43	14.43	12.425 ± 0.08	24.72 ± 0.13
8	M-L	1.50E+03	16.32	15.44	14.24	12.26 ± 0.18	24.78 ± 0.08
9	M-L	1.16E+02	16.52	14.31	17.95	11.43 ± 0.14	28.63 ± 0.06
10	H	2.56E+04	14.17	12.51	13.46	7.39 ± 0.16	20.52 ± 0.13
11	M-L	1.08E+03	18.08	16.03	16.19	9.03 ± 0.03	25.27 ± 0.06
12	M-L	9.45E+02	17.95	16.86	12.94	13.89 ± 1.54	25.48 ± 0.01
13	H	4.32E+04	9.17	8.16	9.67	7.88 ± 0.16	19.73 ± 0.08
14	H	9.63E+04	12.65	12.06	13.64	7.72 ± 0.07	18.52 ± 0.13

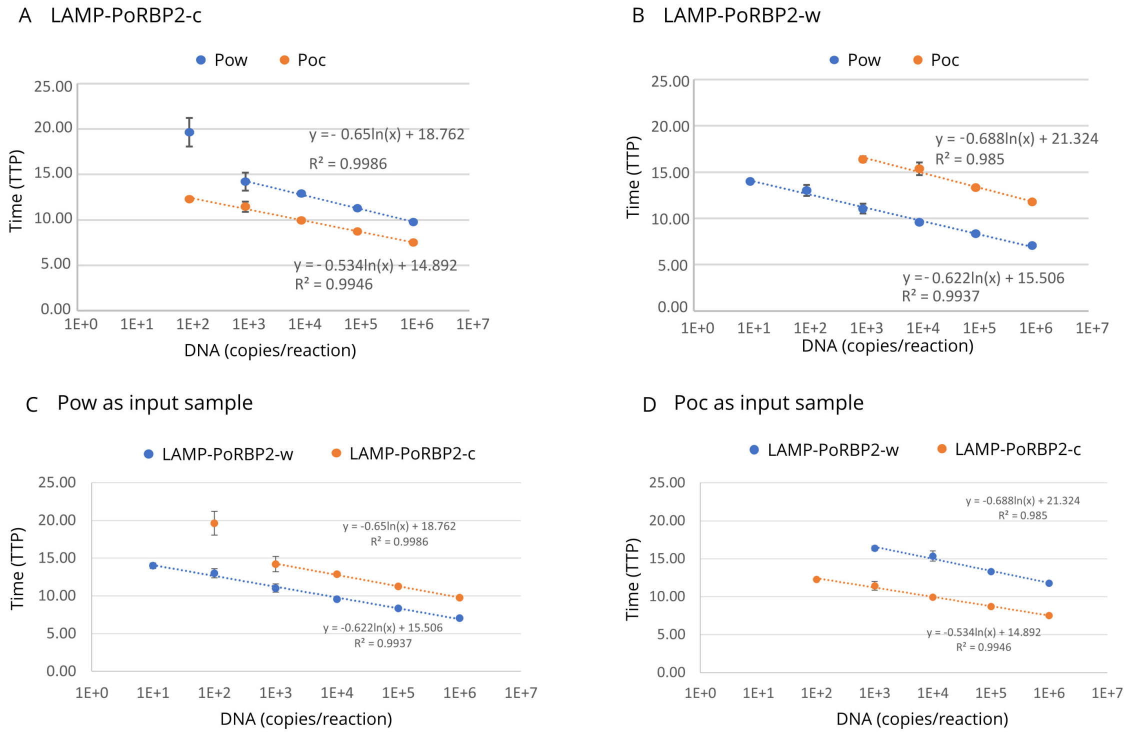

Figure S1. Standard curves of LAMP-PoRBP2-c and LAMP-PoRBP2-w assays. (A) Standard curves of the LAMP-PoRBP2-c assay evaluated with a serial dilution of *P.o. curtisi* and *P.o. wallikeri* synthetic DNA. (B) Standard curves of the LAMP-PoRBP2-w assay evaluated with a serial dilution of *P.o. curtisi* and *P.o. wallikeri* synthetic DNA. C) Standard curves of a serial dilution of *P. ovale wallikeri* using LAMP-PoRBP2-w and LAMP-PoRBP2-c assays. (D) Standard curves of a serial dilution of *P. ovale curtisi* using LAMP-PoRBP2-w and LAMP-PoRBP2-c assays. The specificity of the assays is shown by the earlier TTPs obtained in the specific reactions compared to the non-specific reactions. The assays should be run independently but in parallel and the earliest outcome may indicate the diagnosed species.

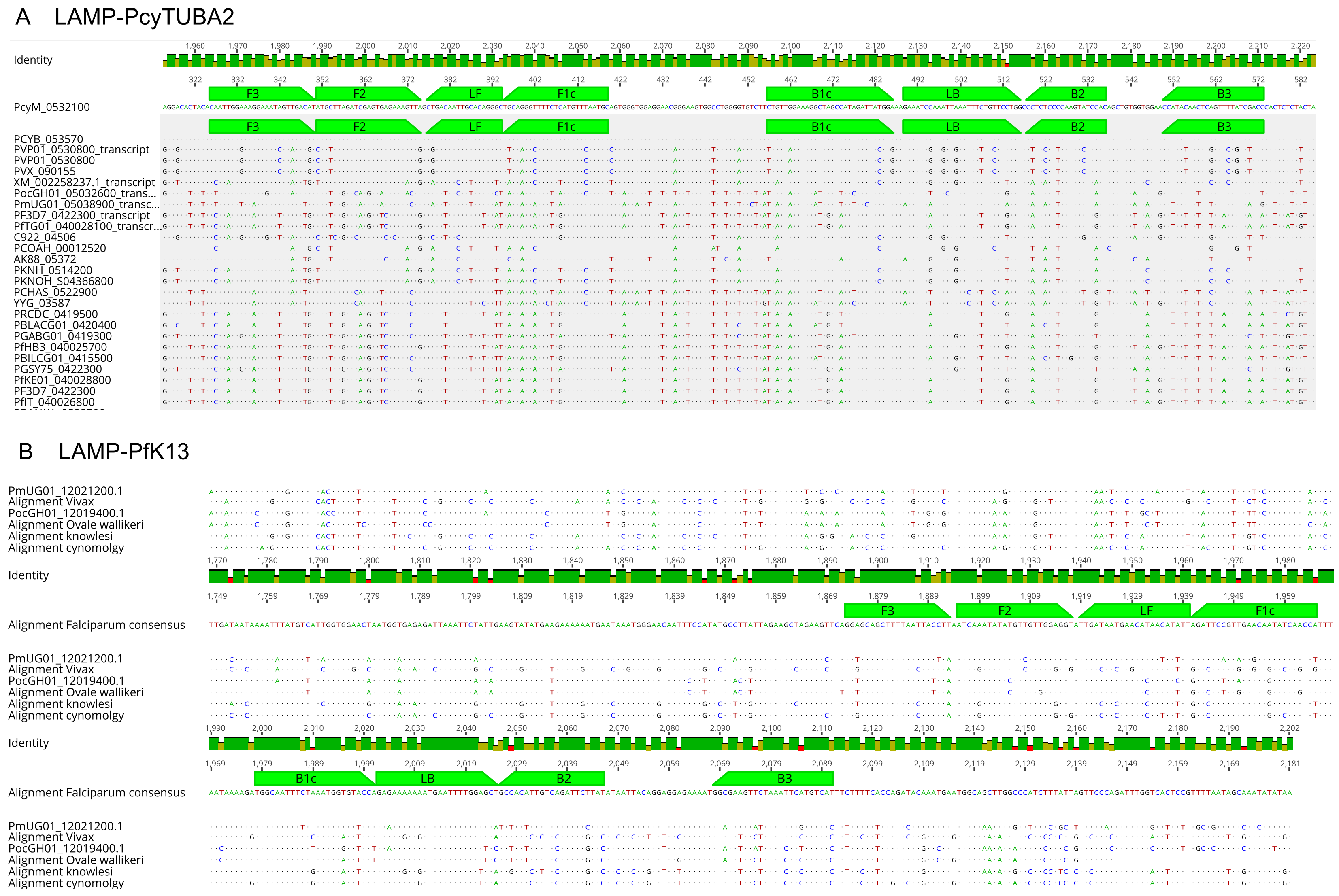

Figure S2. Sequence alignment of all human-infective *Plasmodium* species. (A) Alignment for the design of LAMP-PcyTUBA2. (B) Alignment for the design of LAMP-PcyTUBA2. (C) Alignment for the design of LAMP-PfK13. (D) Alignment for the design of LAMP-PmK13. (E) Alignment for the design of LAMP-PoRBP2-c. (F) Alignment for the design of LAMP-PoRBP2-w. (G) Alignment for the design of LAMP-PvTUBA2.

C LAMP-PfmK13

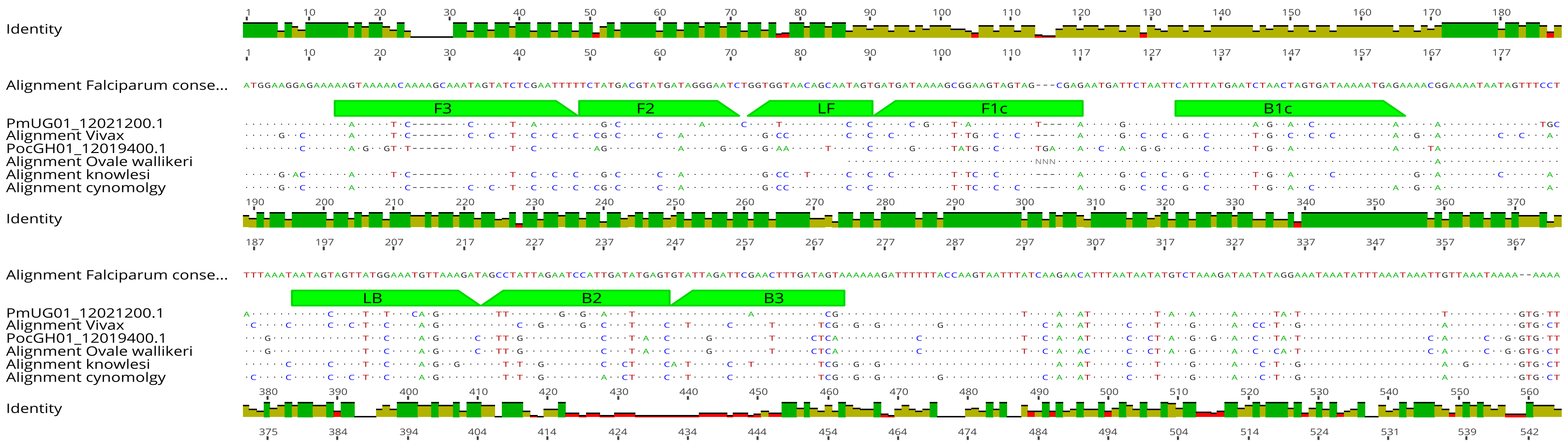

D LAMP-PoRBP2-c

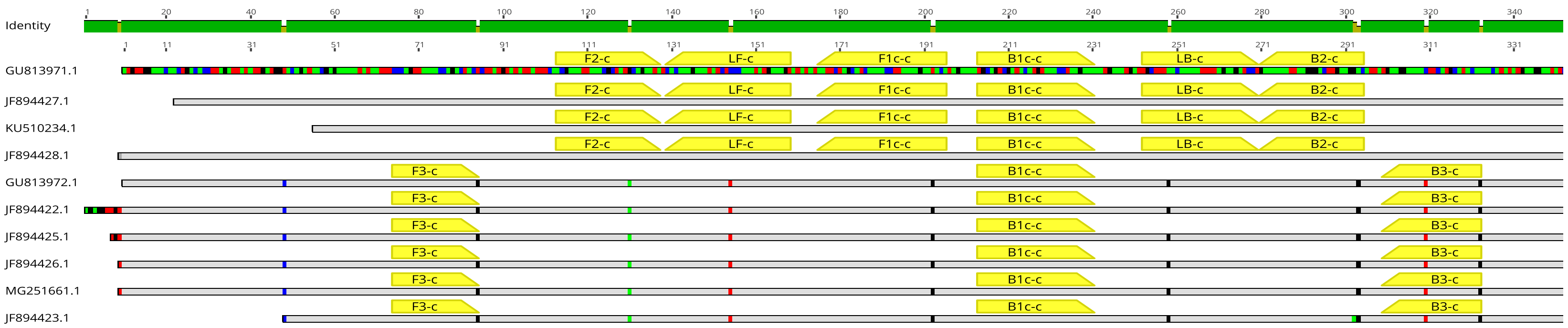

Figure S2. Sequence alignment of all human-infective *Plasmodium* species. (A) Alignment for the design of LAMP-PcyTUBA2. (B) Alignment for the design of LAMP-PcyTUBA2. (C) Alignment for the design of LAMP-PfK13. (D) Alignment for the design of LAMP-PmK13. (E) Alignment for the design of LAMP-PoRBP2-c. (F) Alignment for the design of LAMP-PoRBP2-w. (G) Alignment for the design of LAMP-PvTUBA2.

E LAMP-PoRBP2-w

F LAMP-PvTUBA2

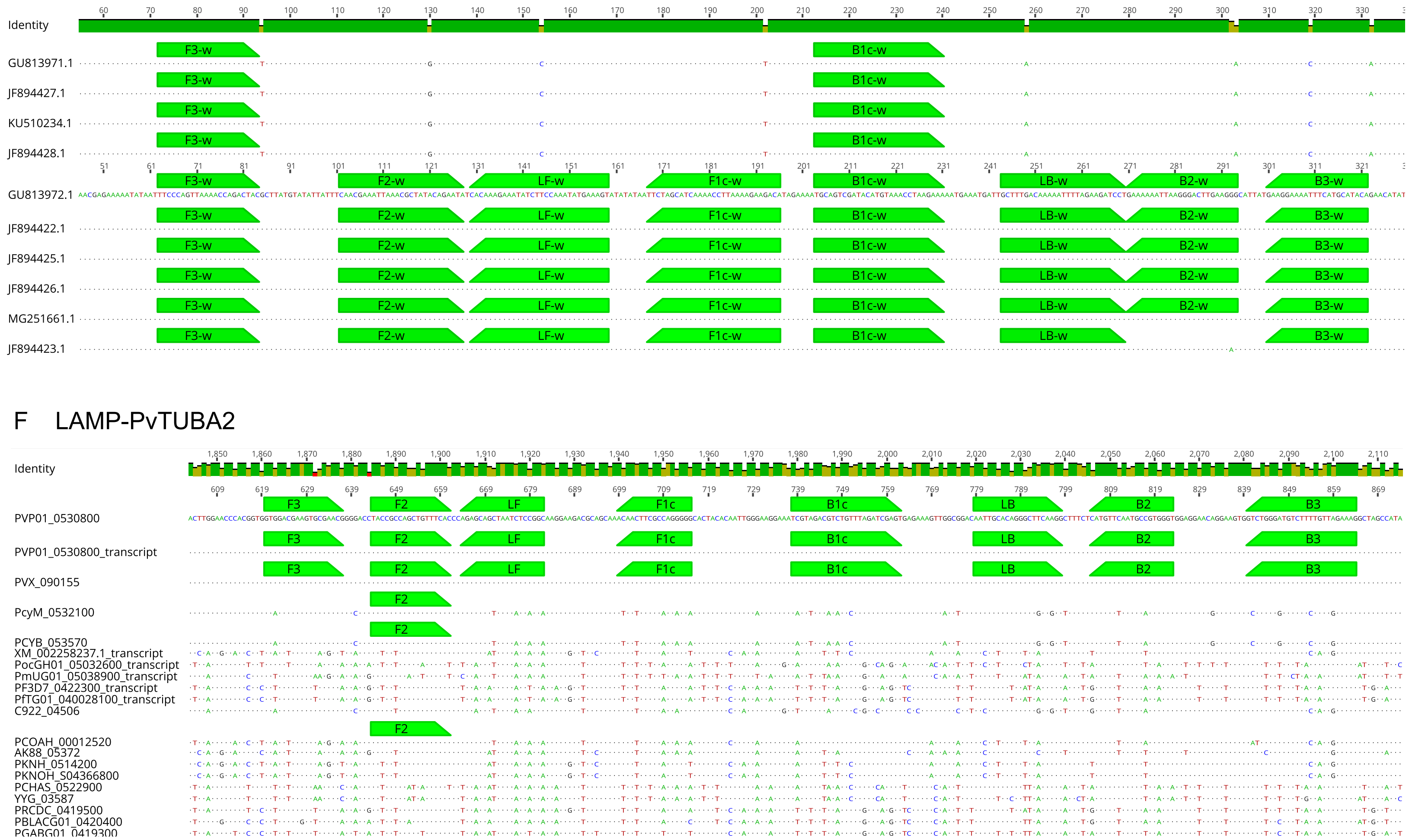

Figure S2. Sequence alignment of all human-infective *Plasmodium* species. (A) Alignment for the design of LAMP-PcyTUBA2. (B) Alignment for the design of LAMP-PfK13. (C) Alignment for the design of LAMP-PmK13. (D) Alignment for the design of LAMP-PoRBP2-c. (E) Alignment for the design of LAMP-PoRBP2-w. (F) Alignment for the design of LAMP-PvTUBA2.

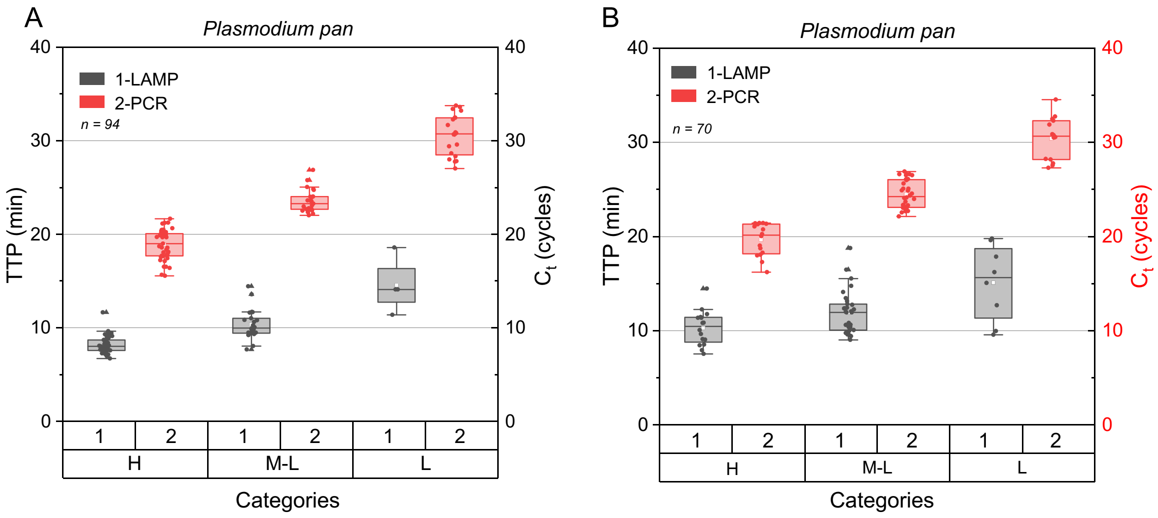

Figure S3. clinical sensitivity and specificity of the assay LAMP-Pan18S with cohort 1 and cohort 2. (A) Boxplot and whisker plots showing the distribution of *Plasmodium-pan* positive samples by PCR-Pan in cohort 1 and LAMP-Pan18S across parasitic loads (H= high, M-L= medium-low, L=low), N = 70. (B) Boxplot and whisker plots showing the distribution of *Plasmodium-pan* positive samples by PCR-Pan and LAMP-Pan18S in cohort 2 across parasitic loads (H= high, M-L= medium-low, L=low), N = 94.

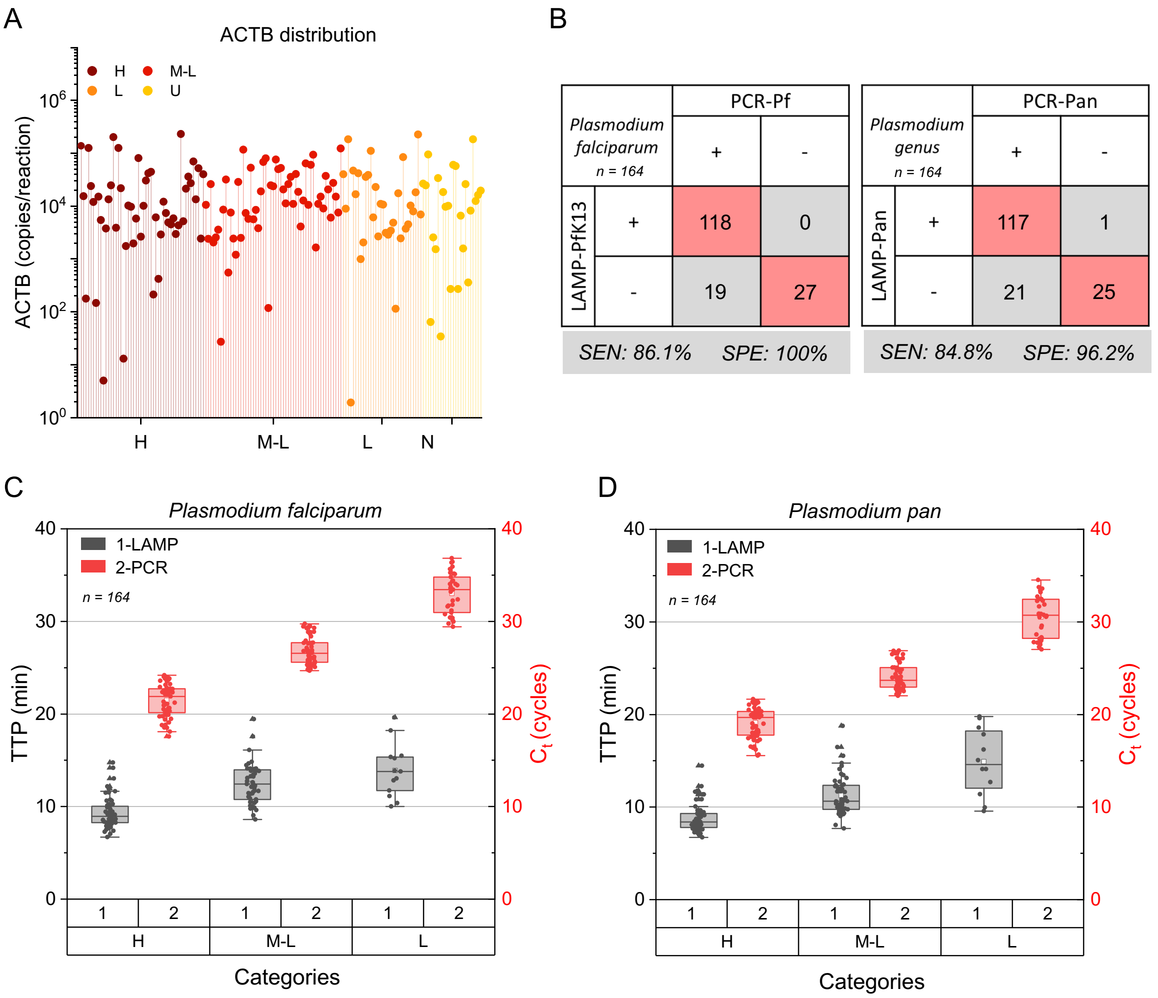

Figure S4. Overall clinical sensitivity and specificity of cohort 1 and 2. (A) Distribution of ACTB concentration across all the samples according to their parasitic load (H= high, M-L= medium-low, L=low, N=negative), N = 164. (B) Clinical sensitivity and specificity matrices of LAMP-PfK13 assay and LAMP-Pan18S versus the gold standard TaqMan qPCR assays PCR-Pf and PCR-Pan, respectively (N = 164).(C) Boxplot and whisker plots showing the distribution of *Plasmodium falciparum* positive samples by PCR-Pf and LAMP-PfK13 across parasitic loads (H= high, M-L= medium-low, L=low, N=negative), N = 164.(D) Boxplot and whisker plots showing the distribution of *Plasmodium-pan* positive samples by PCR-Pan and LAMP-Pan18S across parasitic loads (H= high, M-L= medium-low, L=low, N=negative), N = 164.
